# hnRNPA1 couples nuclear export and translation of specific mRNAs downstream of FGF-2/S6K2 signalling

**DOI:** 10.1093/nar/gku953

**Published:** 2014-10-16

**Authors:** Rajat Roy, Danielle Durie, Hui Li, Bing-Qian Liu, John Mark Skehel, Francesco Mauri, Lucia Veronica Cuorvo, Mattia Barbareschi, Lin Guo, Martin Holcik, Michael J. Seckl, Olivier E. Pardo

**Affiliations:** 1 Division of Cancer, Department of Surgery and Cancer, 1st Floor, ICTEM Building, Hammersmith Hospitals Campus of Imperial College London, Du Cane Road, London W12 0NN, UK; 2 Apoptosis Research Centre, Children's Hospital of Eastern Ontario Research Institute, Ottawa, Ontario, Canada; 3 Department of Biochemistry, Wuhan University, Wuhan, China; 4 Protein Analysis and Proteomics Laboratory, London Research Institute, South Mimms, EN6 3LD, UK; 5 Department of Histopathology, Hammersmith Hospital Campus, Imperial College, London W120NN, UK; 6 Department of Histopathology, S. Chiara Hospital, Trento, Italy

## Abstract

The increased cap-independent translation of anti-apoptotic proteins is involved in the development of drug resistance in lung cancer but signalling events regulating this are poorly understood. Fibroblast growth factor 2 (FGF-2) signalling-induced S6 kinase 2 (S6K2) activation is necessary, but the downstream mediator(s) coupling this kinase to the translational response is unknown. Here, we show that S6K2 binds and phosphorylates hnRNPA1 on novel Ser4/6 sites, increasing its association with BCL-XL and XIAP mRNAs to promote their nuclear export. In the cytoplasm, phosphoS4/6-hnRNPA1 dissociates from these mRNAs de-repressing their IRES-mediated translation. This correlates with the phosphorylation-dependent association of hnRNPA1 with 14-3-3 leading to hnRNPA1 sumoylation on K183 and its re-import into the nucleus. A non-phosphorylatible, S4/6A mutant prevented these processes, hindering the pro-survival activity of FGF-2/S6K2 signalling. Interestingly, immunohistochemical staining of lung and breast cancer tissue samples demonstrated that increased S6K2 expression correlates with decreased cytoplasmic hnRNPA1 and increased BCL-XL expression. In short, phosphorylation on novel N-term sites of hnRNPA1 promotes translation of anti-apoptotic proteins and is indispensable for the pro-survival effects of FGF-2.

## INTRODUCTION

Deregulation of apoptotic cell death is a hallmark of cancer and is involved in the development of resistance to therapy. This is a leading cause of fatalities from common cancers including lung cancer. Several proteins can mediate cell death resistance (1–3) including fibroblast growth factor 2 (FGF-2) (4–6). Indeed, we have shown that FGF-2 signalling leads to the assembly of a multi-protein complex comprising BRaf, protein kinase Cϵ and ribosomal S6 kinase 2 (S6K2) but not S6K1. The activation of S6K2 occurs in a stepwise manner initiated by phosphorylation of the three proline-directed serines in the auto-inhibitory domain, Ser-410, Ser-417 and Ser-423 downstream of MEK/ERK signalling. Subsequently, phosphorylation of Ser-370 then enables phosphorylation of Thr-388 by the mTORC1 complex followed by that of Thr-228 by PDK1 (7). Activated S6K2 then enhances the translation of anti-apoptotic proteins such as BCL-XL and X chromosome-linked inhibitor of apoptosis (XIAP) (4). Translation of these mRNAs under conditions of cellular stress including anti-cancer therapies is mediated by an internal ribosomal entry site (IRES) located in the 5′ untranslated region (UTR) (8–10). However, we only have limited understanding of the modulators of IRES-based translation downstream of S6K2 activation (11). S6K2 shuttles between the nucleus and the cytoplasm in response to growth factor signalling. In addition to its diffuse nuclear localization, a proportion of S6K2, but not S6K1, has been shown to co-localize with CTR453 and γ-tubulin at the level of the centrosome (7).

The hnRNP family comprises at least 20 members termed hnRNPA1 to hnRNPU (12). These proteins are mainly located in the nucleus, associated with pre-mRNA to influence their splicing, metabolism and transport with some hnRNPs reported to shuttle between the nucleus and the cytoplasm (12). Among these, hnRNPA1 was shown to contain a novel nuclear localisation signal, called M9, which enables both its nuclear entry and exit (13). Interestingly, interfering with hnRNPA1 shuttling, and the resulting accumulation of this protein in the cytoplasm, prevents BCL-XL and XIAP expression in several cell systems (9,10,14). Moreover, when hnRNPA1 is bound to these mRNAs, it suppresses IRES-mediated translation (9,10,15–17). However, the regulation of this hnRNPA1 activity downstream of cellular signalling is not understood.

Here, we identified hnRNPA1 as a distinct substrate for S6K2 downstream of FGF-2 signalling that is crucial for the anti-apoptotic function of this pathway. We found that FGF-2 signalling promotes the cycling of hnRNPA1 between the nucleus and the cytoplasm through a series of stepwise post-translational modifications. FGF-2 stimulation and S6K2 activation lead first to the phosphorylation of hnRNPA1 on a novel site (Ser4) that promotes the binding of this protein to BCL-XL and XIAP mRNAs. Then, following association with the export factor Nxf-1, hnRNPA1/mRNA complexes are exported from the nucleus in a MEK-dependent fashion. Finally, once in the cytoplasm, phosphorylated hnRNPA1 interacts with 14-3-3σ and θ leading to its sumoylation on K183. This last event is necessary for the re-import of hnRNPA1 and subsequent shuttling cycles as sumo-deficient mutants for this site accumulate in the cytoplasm. In short, our work demonstrates a previously undescribed mechanism through which FGF-2 signalling increases the export of BCL-XL and XIAP mRNAs out of the nucleus and promotes their subsequent translation. Targeting the enzymes that regulate these post-translational modifications may provide a novel and efficient way to silence this pro-survival signalling mechanism in the treatment of cancer.

## MATERIALS AND METHODS

### Reagents and antibodies

See Supplementary Materials.

### Plasmids

pCEMM-GS-TAP vector was a kind gift from Dr A. Bauch (18). pCMV6-hnRNPA1-FLAG was from Origene and mutations (S4AS6A, S4DS6D and K183R) introduced by directed mutagenesis. The Myc-MBP-M9M construct was from Dr Y. M. Chook (19). HA-SUMO1 construct was obtained via Addgene (21154) from Dr J. Yuan (20).

### Cell culture

H510 and HEK293 cells were maintained in RPMI-1640 or Dulbecco's modified Eagle's medium, respectively, with 10% foetal calf serum (FCS). Prior to FGF-2 treatment, cells were starved in FCS-free media (21) for 3 days (H510) or overnight in 0.5% FCS media (HEK293). DNA constructs were transfected using Attractene and siRNAs (20 nM) with Lipofectamine RNAiMAX (according to manufacturer's protocol).

### Dimethyl-labelling quantitative phosphoproteomics

H510 cells grown in serum-free medium (21) were treated ± 0.1 ng/ml FGF-2 or 10 ng/ml SCF for 10 min at 37°C, 5% CO_2_. Stimulated and unstimulated samples were then subjected to differential dimethyl-labelling using heavy or light formaldehyde as previously described (22). Phosphopeptides were enriched using immobilized metal affinity chromatography method (23) and samples analysed using LC-MS/MS QSTAR ELITE mass spectrometer (Applied Biosystems).

### TAP-tag purifications

S6K2 and hnRNPA1 open reading frames (ORFs) were cloned into the GS-TAP vector by gateway technology (Invitrogen) to produce Protein G and streptavidin binding peptide N-terminal fusion constructs. HEK293 cells expressing TAP-S6K2 or TAP-hnRNPA1 were treated ± FGF-2 for 10 min, lysed in LB (50 mM Tris/HCl, pH 7.5, 125 mM NaCl, 5% glycerol, 0.2% NP-40, 1.5 mM MgCl_2_, 1 mM DTT, 25 mM NaF, 1 mM Na_3_VO_4_, 1 mM EDTA and protease inhibitors) and lysates cleared by centrifugation. These were incubated with rabbit IgG agarose (Sigma) (2 h, 4°C). Bound proteins were washed three times in LB and twice with TEV cleavage buffer (10 mM Tris/HCl, pH 7.5, 150 mM NaCl, 0.5 mM EDTA, 0.2% NP-40) and eluted by incubating with 100U of TEV protease (2 h, 16°C). TEV cleaved lysates were incubated with Streptavidin beads (Pierce) (2 h, 4°C) and washed four times with cleavage buffer supplemented with protease/phosphatase inhibitors. Bound proteins were eluted by boiling in Laemmli buffer and separated on a 4–15% sodium dodecyl sulphate-polyacrylamide gel electrophoresis (SDS-PAGE). Protein lanes were excised as 1 mm gel slices and digested with trypsin. Peptides were analysed using an orthogonal acceleration quadrupole Tof mass spectrometer (SYNAPT HDMS, Waters, UK). Liquid Chromatography/Mass spectrometry (LC/MS/MS) data were then searched against a non-redundant protein database (UniProt 12.4) using the Mascot search engine (Matrix Science, UK) and analysed using the Scaffold software.

### Phosphopeptide arrays

14-aa long overlapping peptides covering hnRNPA1 were synthesized and spotted on nitrocellulose membranes (Supplementary Figure S5). The arrays were washed extensively in kinase buffer (20 mM Tris pH 8, 20 mM MgCl_2_, 2 mM MnCl_2_) prior to *in vitro* kinase assay with recombinant kinases, ^32^P-γATP and cold ATP in kinase buffer (15 min, 37°C). The reactions were stopped with ethylenediaminetetraaceticacid (EDTA) and the arrays washed once with 1M NaCl (20 min), followed by 1% SDS (20 min) and 15 washes with 0.5% phosphoric acid. Following a final wash with 96% ethanol, the arrays were autoradiographed.

### Subcellular fractionation

Cells were re-suspended in fractionation buffer (10 mM HEPES pH 7.6, 3 mM MgCl_2_, 10 mM KCl, 5% (v/v) glycerol, 1% Triton-X100, protease/phosphatase inhibitors) on ice for 10 min and then centrifuged at 250 g (5 min, 4°C). The supernatant was further centrifuged (18 000 g, 10 min) to obtain a cleared cytoplasmic fraction. The pellet was washed in ice-cold wash buffer (10 mM HEPES pH 7.6, 1.5 mM MgCl_2_, 10 mM KCl, protease/phosphatase inhibitors) (250 g, 5 min, 4°C) prior to re-suspension in nuclear extraction buffer (20 mM HEPES pH 7.6, 1.5 mM MgCl_2_, 420 mM NaCl, 25% (v/v) glycerol, 0.2 mM EDTA, protease/phosphatase inhibitors) and sonicated (30 min in ice-cold water). The lysate was then centrifuged (18 000 g, 10 min, 4°C) and the supernatant transferred to fresh tubes as the crude nuclear extract.

### Co-immunoprecipitation

Antibodies were conjugated to Protein A or G magnetic beads (Dyanal). When following subcellular fractionation, the cytoplasmic fraction was mixed with equal volume of IP dilution buffer (90 mM HEPES pH 7.6, 240 mM Nacl, 1% NP40, 4 mM EDTA, protease/phosphatase inhibitors) and the subsequent washes performed with 0.5X IP dilution buffer. For SUMO immunoprecipitations, *N*-ethyl malaeimide (20 mM) was added to the IP and wash buffers.

### RNA immunoprecipitation

RNA immunoprecipitation (RNA-IP) was performed as described in (24). Antibody-conjugated magnetic beads (A or G) were used to immunoprecipitate hnRNPA1 from the cytoplasmic or nuclear extracts; the associated mRNA was reverse transcribed into cDNA and analysed by quantitative polymerase chain reaction (qPCR). For primer sequences see Supplementary Materials. Absolute quantification of mRNAs was performed in SDS 2.4 software (ABI instruments) using a standard curve from serial dilution of the input. Samples were normalised to their corresponding input and presented as a percentage of input as previously described (25). The levels of immunoprecipitated protein and the amounts of input RNA corresponding to the RNA-IPs in Figure 5 are presented in Supplementary Figure S7.

**Figure 1.**
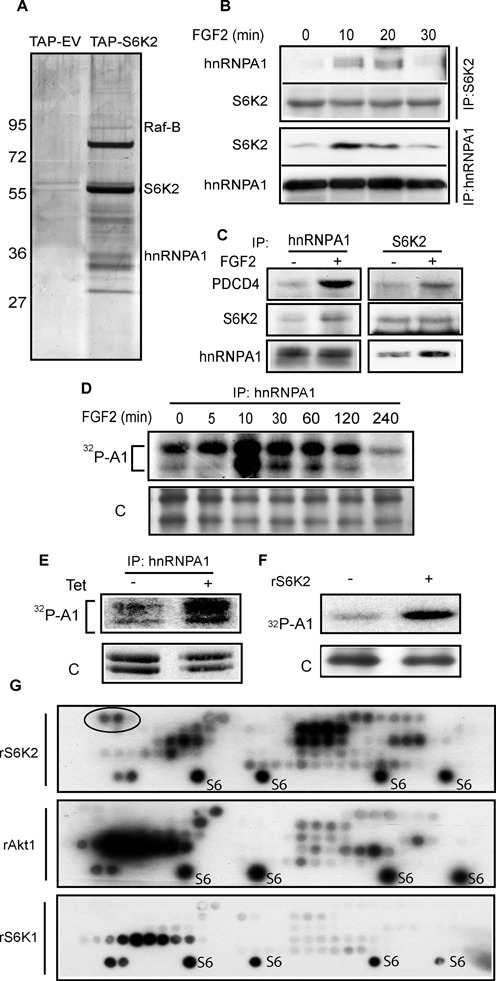
hnRNPA1 interacts with and is phosphorylated by S6K2. (**A**) HEK293 cells expressing TAP-tagged S6K2 were subjected to Tandem Affinity Purification, proteins separated by SDS-PAGE and the silver stained bands identified by MS. (**B** and **C**) S6K2 or hnRNPA1 were immunoprecipitated from H510 (B) or HEK293 (C) cells treated with FGF-2 and associated proteins detected by western blot. (**D**) HEK293 cells were labelled with ^32^Pi phosphate prior to treatment with FGF-2 and hnRNPA1 immunoprecipitation. The samples were separated by SDS-PAGE and autoradiographed. (**E**) HEK293 cells expressing tetracycline-inducible kinase-active S6K2 were labelled with ^32^Pi phosphate and hnRNPA1 immunoprecipitation performed post treatment ± doxycline (Dox). The samples were separated on SDS-PAGE prior to autoradiography. (**F**) Recombinant (r) hnRNPA1 and S6K2 were used in an *in vitro* kinase (IVK) assay in presence of ^32^P γATP, prior to SDS-PAGE and autoradiography. (**D, E** and **F**) ‘C’; silver stain control. (**G**) hnRNPA1 peptide arrays were spotted on nitrocellulose membrane and IVK assays performed with rS6K2, rS6K1 or rAkt1 in presence of ^32^P γATP prior to autoradiography. Ribosomal S6 peptide (S6) was used as a positive control. Two peptides uniquely phosphorylated by S6K2 are circled. All experiments are representative of at least three independent repeats. See also Supplementary Tables S1–S4 and Supplementary Figure S1.

**Figure 2.**
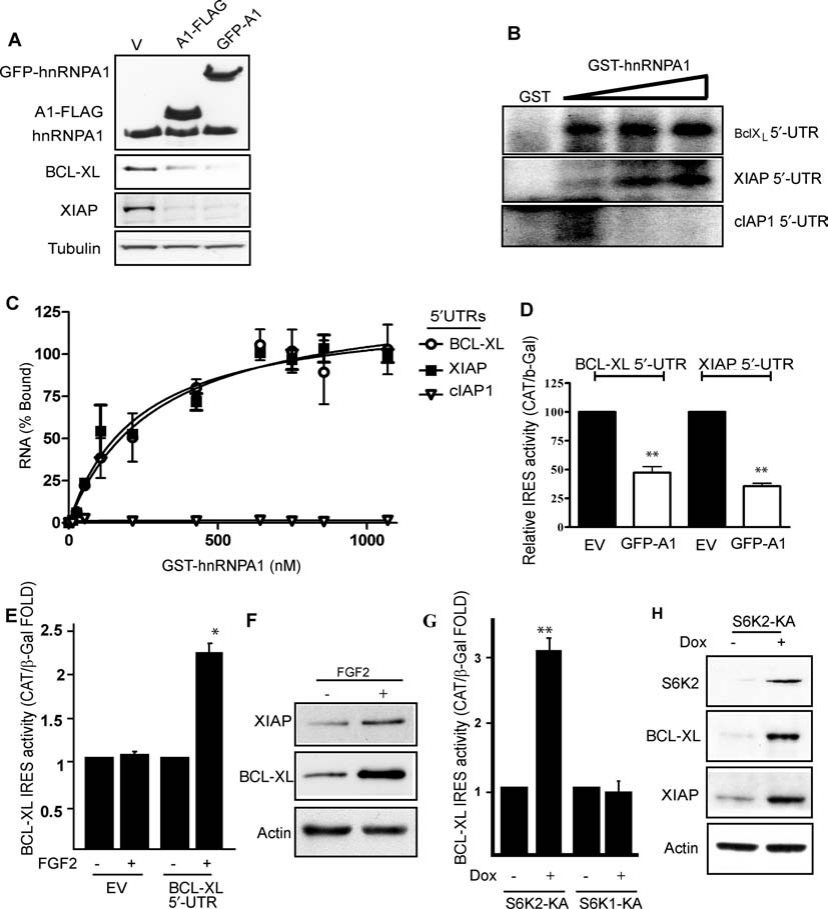
hnRNPA1 binds to BCL-XL, XIAP transcripts *in vitro* and regulates their translation. (**A**) N-term GFP- and C-term FLAG-tagged hnRNPA1 were transiently expressed in HEK293 cells and western blotting (WB) performed for the indicated proteins 72 h later. (**B** and **C**) Recombinant GST-hnRNP A1 was incubated in the presence of ^32^P-labelled, *in vitro* transcribed BCL-XL, XIAP or cIAP1 (negative control) 5′-UTR RNAs and UV crosslinked. (B) RNA–protein complexes were analysed by SDS-PAGE/autoradiography. GST was used as a negative control. (C) Increasing concentrations of GST-hnRNPA1 were used to bind the UTRs and the signal from nitrocellulose filter binding assays quantified by beta counter. (**D**, **E** and **G**) Bicistronic DNA constructs containing XIAP or BCL-XL IRES elements were co-transfected into (D) HEK293 cells along with GFP (EV) or GFP-hnRNPA1 (GFP-A1) expressing plasmids, (E) transfected into HEK293 cells treated ± FGF-2 for 4 h or (G) into HEK293 cells stably expressing tetracycline-inducible kinase-active (KA) S6K1 or 2 treated overnight ± doxycline (Dox). IRES activity was measured as ratio of CAT to β-gal expression. (**F** and **H**) HEK293 cells (F) or S6K2-KA HEK293 cells (H) were stimulated ± FGF-2 for 4 h or overnight ± Dox, respectively, and samples analysed by WB for the indicated proteins. (C, D, E and G) Results are the average of triplicates ± SEM. Student's t-test: *P < 0.05; **P < 0.01.

**Figure 3.**
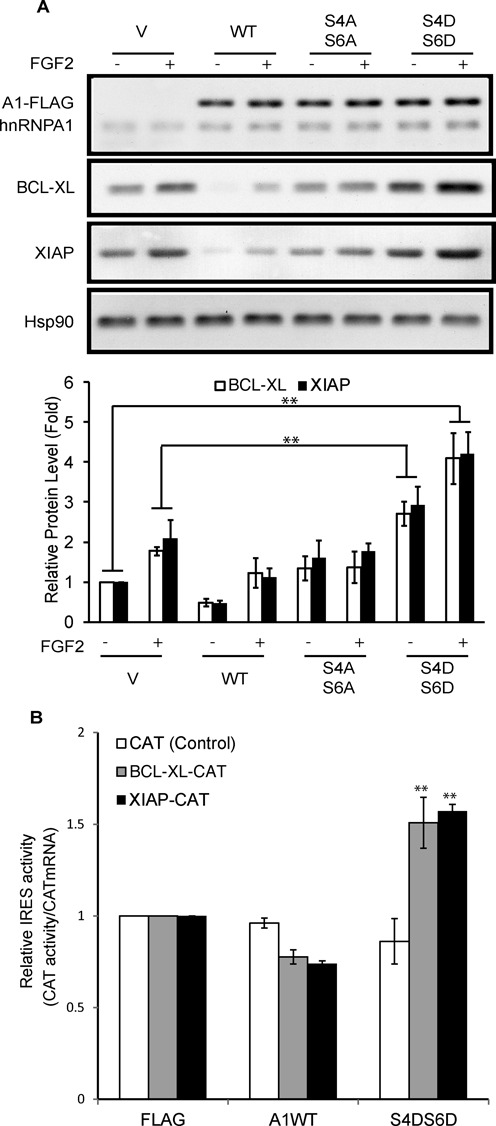
Phosphorylation of Ser4/6 on hnRNPA1 regulates BCL-XL and XIAP expression. (**A** and **B**) HEK293 cells were transiently transfected with wild-type (WT), mutant (S4AS6A or S4DS6D) hnRNPA1 or vector alone (V). (A) Cells were treated ± FGF-2 for 4 h prior to SDS-PAGE/western blotting for the indicated proteins. Lower panel: the BCL-XL and XIAP signals were quantified and normalized to that of HSP90. (B) Cells were subsequently transfected with reporter mRNAs driving CAT from the BCL-XL or XIAP IRES. CAT activity was normalised to the amount of injected mRNA. CAT alone mRNAs were used as control. Results are average of (A, lower panel) three independent experiments ± SEM or (B) triplicates ± SEM. Student's t-test: *P < 0.05; **P < 0.01.

**Figure 4.**
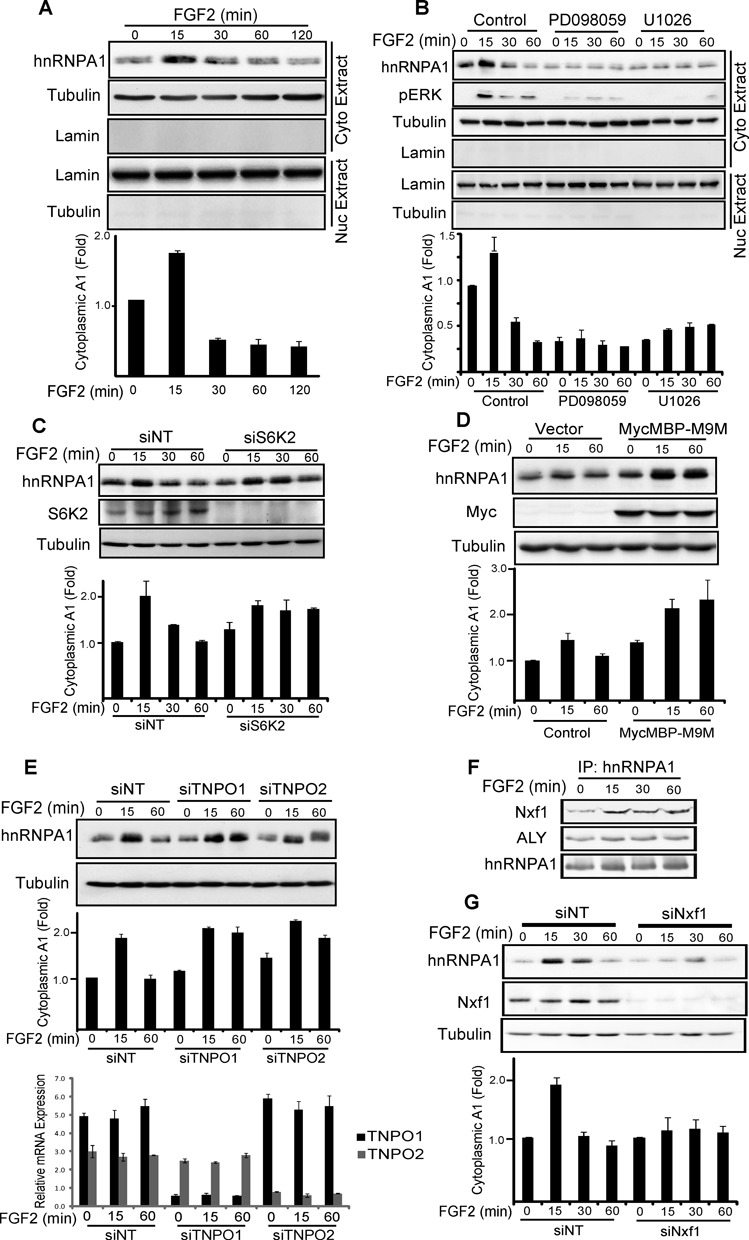
hnRNPA1 shuttles between cytoplasm and nucleus in response to FGF-2. HEK293 cells alone (**A**), pre-treated with the MEK inhibitors PD098059, U1026 or DMSO alone for 1 h (**B**), transfected with siRNA for S6K2 (**C**), TNPO1 and 2 (**E**) or NXF1 (**G**) or the karyopherin inhibitor MycMBP-M9M vector or vector alone (**D**) were treated ± FGF-2 for the indicated time. The cytoplasmic fraction was analysed by SDS-PAGE/western blotting (WB) for the indicated proteins. S6K2 (C), TNPO1 and 2 (E) or NXF1 (G) knockdown were confirmed by SDS-PAGE/WB (C and G) or qPCR (E, lower panel). (A–E and G Lower panel) The hnRNPA1 signal was quantified and normalized to that of tubulin. (**F**) Endogenous hnRNPA1 was immunoprecipitated from HEK293 cells treated ± FGF-2. Immunoprecipitates were analysed by SDS-PAGE/WB for the indicated proteins. (A–G) Results are representative of at least three independent experiments. Bar graphs are average from replicate experiments ± SEM. See also Supplementary Figure S2.

**Figure 5.**
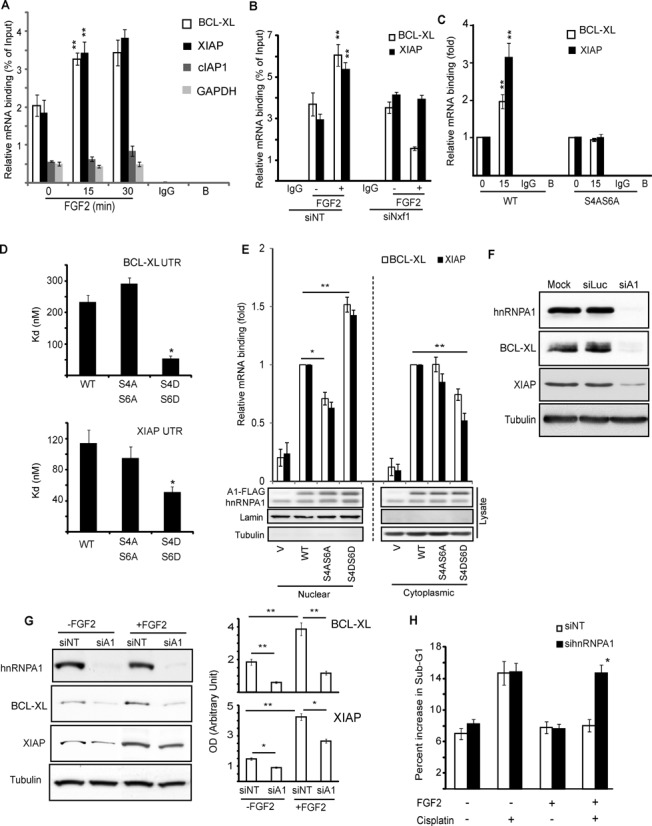
hnRNPA1 mediates nuclear export of BCL-XL and XIAP mRNA in response to FGF-2. (**A**) HEK293 cells, (**B**) HEK293 cells transfected with Nxf1 siRNA or non-targeting control (NT) siRNA or (**C**) HEK293 cells expressing wild-type or S4AS6A mutant hnRNPA1 were treated with FGF-2 for the indicated durations (A and C) or 30 min (B). The cytoplasmic fractions were subjected to RNA immunoprecipitation (RNA-IP) with hnRNPA1 antibody and the associated RNA analysed by quantitative PCR (qPCR) using primers directed against the indicated transcripts. (**D**) Increasing concentrations of recombinant wild-type (WT), S4AS6A or S4DS6D mutant hnRNPA1 were incubated with ^32^P-labelled, *in vitro* transcribed XIAP or BCL-XL 5′-UTR, nitrocellulose filter binding assays performed and binding quantified as in Figure 2C. Kd values were calculated from three independent experiments using non-linear one-site specific binding. (**E**) HEK293 cells were transiently transfected with WT, S4AS6A, S4DS6D hnRNPA1 or vector alone (V) and subcellular fractionation was performed 48 h later to separate the nuclear and cytoplasmic fractions. These were subjected to RNA-IP and qPCR as in (A). Results are expressed as average fold changes from triplicate ± SEM with cells expressing WT-hnRNPA1 as reference. (**F**) HEK293 cells were transfected with hnRNPA1 (siA1) or luciferase (siLuc) control siRNAs or mock transfected (transfection reagent alone). Cell lysates were analysed by SDS-PAGE/western blotting (WB). (**G**) HEK293 cells transfected ± hnRNPA1 or non-targeting (siNT) siRNAs and treated ± FGF-2 for 4 h followed or not by treatment with cisplatin for 48 h were (G) analysed by SDS-PAGE/WB for their BCL-XL and XIAP levels or (**H**) analysed by flow cytometry following PI staining for the appearance of a Sub-G1 (apoptotic) population. Results are average of triplicates ± SEM. (G) Left panel: average of intensities for BCL-XL and XIAP bands from three experiments were normalized to that of tubulin and plotted ± SEM. (A–H) Results representative of at least three independent experiments. Student's t-test: *P < 0.05; **P < 0.01.

### Synthetic RNA preparation

Radioactive BCL-XL or XIAP 5′-UTR RNAs for UV crosslinking and nitrocellulose filter binding assays were prepared by *in vitro* transcription. KOD PCR was used to generate DNA templates for *in vitro* transcription. For T7 primer sequences see Supplementary Materials. Gel-purified products (Ultra Clean^TM^ 15, MO BIO Laboratories) were *in vitro* transcribed in the presence of ^32^P UTP (10–25 mCi/ml) using Maxiscript kit (Ambion) and gel purified. Full-length RNA bands were cut out and eluted in water overnight before purification (RNA miniprep columns; Agilent Technologies). Monocistronic RNA reporters (mono BCL-XL or monoCAT) containing the chloramphenicol acetyltransferase (CAT) reporter gene used in RNA transfections were prepared as described previously (26).

### CAT reporter analysis

Determination of CAT expression from transfected *in vitro* synthesized RNA reporter constructs was as previously described (26).

### UV crosslinking of RNA–protein complexes

RNA–protein UV crosslinking and nitrocellulose filter binding assays were done as previously (10).

### Immunohistochemistry

Tissue microarray (TMA) samples from lung cancer patients (*n* = 204) or breast cancer patients (*n* = 194) were stained for S6K2, hnRNPA1 and BCL-XL and staining scores calculated as described earlier (27,28). Please see Supplementary Methods for further details.

## RESULTS

### S6K2 interacts with and phosphorylates hnRNPA1

We have previously shown that S6K2 mediated the pro-survival activity of FGF-2 by promoting the translation of the anti-apoptotic proteins BCL-XL and XIAP in several cell types including H-510 SCLC and HEK-293 cells (4). Here, two independent strategies were used to identify proteins that mediate this translational effect. The first approach employed quantitative phosphoproteomic analysis of H510 cells (Supplementary Table S1) and the second utilised Tandem Affinity Purification (TAP) using S6K2 as bait in HEK293 cells (Figure 1A and Supplementary Table S2). Both experimental approaches were performed with and without FGF-2 stimulation. To help focus on changes linked to the pro-survival activity of FGF-2, we repeated the quantitative phosphoproteomic experiments in the presence of an alternate stimulus, stem cell factor (SCF) (Supplementary Table S3), which is involved in the proliferation (29–31), but not survival, of lung cancer cells (our unpublished observations). These experiments revealed several RNA binding proteins among which PDCD4 (11) and hnRNPA1 interacted with S6K2 and were specifically phosphorylated in an FGF-2-dependent manner (Figure 1A, Supplementary Tables S1 and S2 and Supplementary Figure S1A). We have recently shown that PDCD4 is involved in FGF-2/S6K2 signalling (11) but the role of hnRNPA1 in this process is unknown. We initially confirmed that S6K2 and hnRNPA1 could associate in an FGF-2-dependent fashion using co-immunoprecipitation in both H510 (Figure 1B) and HEK293 (Figure 1C) cells. Similarly, hnRNPA1 and S6K2 also co-immunoprecipitated with PDCD4, suggesting that these three proteins may be part of the same molecular complex (Figure 1C). The association of S6K2 with hnRNPA1 was time dependent peaking 10 min following FGF-2 stimulation (Figure 1B). We also confirmed that FGF-2 could induce the phosphorylation of hnRNPA1. Indeed, both hnRNPA1 splice variants were phosphorylated following FGF-2 stimulation of ^32^P_i_ phosphate labelled H510 cells (Figure 1D). To investigate whether S6K2 could be the kinase mediating hnRNPA1 phosphorylation, we used HEK293 cells expressing a tetracycline-inducible kinase-active (KA) version of S6K2 (4). This revealed that increased S6K2 activity led to phosphorylation of hnRNPA1 in ^32^P_i_ labelled cells (Figure 1E). This was a direct effect as an *in vitro* kinase assay performed in the presence of recombinant hnRNPA1, recombinant active S6K2 and ^32^P-γATP resulted in hnRNPA1 phosphorylation (Figure 1F). AGC family kinases such as S6K2 target the consensus sequence R–X–R–X–X–S/T (32), but additional specificity of individual family members is poorly documented. We therefore next investigated whether S6K2 targeted a specific phosphosite(s) in hnRNPA1 distinct from those that may be phosphorylated by the closely related S6K1 and the prototypical AGC kinase, AKT. Peptide arrays fully covering hnRNPA1 as a series of overlapping 14 amino acid sequences were printed onto nitrocellulose and subjected to equal units of activity of recombinant S6K2, S6K1 or AKT. Figure 1G shows that while all three kinases phosphorylated various sites in hnRNPA1, some peptides were specifically targeted by S6K2. These included a region with the minimum sequence 1-M S K S E S P-7 situated at the N-terminus of the protein (Figure 1G, circled). As both Ser4 and Ser6 had been identified in our quantitative phosphoproteomic approach (Supplementary Table S1), we decided to focus on understanding the biological role of these post-translational modifications. However, before attempting this we wished to confirm the relevance of hnRNPA1 in controlling BCL-XL and XIAP translation.

### hnRNPA1 binds BCL-XL and XIAP mRNAs and represses their translation

We have previously shown that hnRNPA1 binds the 5′-UTR of XIAP mRNA and inhibits the translation of this protein (10). We confirmed that the overexpression of hnRNPA1 decreased BCL-XL protein levels in HEK293 cells (Figure 2A). Using radio-labelled RNA binding assays, we showed that hnRNPA1 bound to the 5′-UTR of BCL-XL and XIAP mRNAs with comparable affinity (Figure 2B and C), but not to the 5′-UTR of the related IAP, cIAP-1. The 5′-UTRs of BCL-XL and XIAP mRNAs differ from that of cIAP-1 in that they contain IRESs that regulate their cap-independent translation (10). Hence, we tested whether the ability of hnRNPA1 to bind the IRES of BCL-XL and XIAP could control the translation from these IRESs. HEK293 cells were transfected with a bicistronic vector (11) where CAT expression was driven by either the BCL-XL or XIAP 5′-UTR (cap-independent), while beta-galactosidase expression (used for normalisation of CAT activity) was cap-dependent. In agreement with the effects observed on BCL-XL and XIAP levels (Figure 2A), Figure 2D shows that co-expression of hnRNPA1 resulted in a drop of cap-independent translation from both the XIAP and BCL-XL IRESs. Conversely, stimulation of HEK293 cells containing the BCL-XL bicistronic vector with FGF-2 for 4 h resulted in enhanced cap-independent translation (Figure 2E) that correlated with increased levels of BCL-XL and XIAP proteins (Figure 2F). Similarly, transfection of the BCL-XL bicistronic vector into HEK293 cells containing tetracycline-inducible KA-S6K1 or 2 showed that while induction of KA-S6K1 did not regulate cap-independent translation, KA-S6K2 increased IRES-driven CAT activity (Figure 2G). Here again, this correlated with the ability of KA-S6K2 to upregulate BCL-XL and XIAP protein levels (Figure 2H). Together, these results confirmed that hnRNPA1 binds to the 5′-UTRs of BCL-XL and XIAP mRNAs to repress IRES-mediated translation and suggest that KA-S6K2 might reverse this process.

### Phosphorylation of hnRNPA1 on Ser4 and 6 regulates BCL-XL and XIAP translation

The preceding findings suggested that FGF-2/S6K2-induced phosphorylation of hnRNPA1 on Ser4 and/or 6 might enhance translation of BCL-XL and XIAP. To test this we generated wild-type (WT), non-phosphorylatable (S4A-S6A) and phospho-mimetic (S4D-S6D) versions of hnRNPA1. HEK-293 cells expressing comparable amounts of these proteins were treated with or without FGF-2 and analysed by western blotting. We confirmed that overexpression of WT-hnRNPA1 reduced basal levels of BCL-XL and XIAP (Figure 3A). In contrast, the S4A-S6A mutant failed to repress the basal expression of these proteins but did prevent their induction by FGF-2. However, the S4D-S6D hnRNPA1 mutant increased the basal BCL-XL and XIAP levels by over 2-fold (Figure 3A) and did not prevent their further induction by FGF-2. We therefore hypothesized that the Ser4/6 phosphorylated hnRNPA1 could mediate cap-independent upregulation of BCL-XL and XIAP induced by FGF-2. In agreement with this, the phospho-mimetic S4D-S6D hnRNPA1 mutant increased cap-independent translation driven by the 5′-UTRs of BCL-XL and XIAP in HEK293 cells transfected with the corresponding monocistronic vector (Figure 3B). Together, these results suggest that FGF-2-induced phosphorylation of hnRNPA1 on Ser4/6 leads to increased cap-independent translation of BCL-XL and XIAP.

### FGF-2 mediates nucleo-cytoplasmic shuttling of hnRNPA1

Protein translation occurs in the cytoplasm (33) while hnRNPA1 is predominantly nuclear (34). We therefore investigated whether FGF-2 signalling could mediate the effects of hnRNPA1 on translation by controlling its subcellular distribution. Cellular fractionation studies in HEK293 cells revealed a peak of cytoplasmic hnRNPA1 15 min following FGF-2 stimulation, followed by a drop of the levels of this protein below background levels at later time points (Figure 4A and Supplementary Figure S2A). The cytoplasmic accumulation of hnRNPA1 was dependent on MEK/ERK signalling as this was blocked by the MEK inhibitors PD098059 or U1026 (Figure 4B). However, the downstream target of MEK/ERK, S6K2 (21), did not affect the increase in cytoplasmic hnRNPA1 levels as siRNA-mediated silencing of this kinase failed to block the peak of hnRNPA1 at 15 min (Figure 4C). Nevertheless, S6K2 silencing prevented the subsequent drop in hnRNPA1 levels (Figure 4C). The rapid changes in cytoplasmic levels of hnRNPA1 could be due to nucleo-cytoplasmic shuttling. As most cellular hnRNPA1 resides in the nucleus with very little being present in the cytoplasm (Supplementary Figure S2A) it was impossible to visualise either by immunofluorescence or biochemical techniques an early reduction or late increase in the nuclear hnRNPA1 levels upon FGF-2 treatment. An alternative explanation for the effects seen could reside in altered hnRNPA1 degradation which might for example be enhanced at 30 min to account for the reduction in cytoplasmic levels. However, neither inhibition of the proteasome using MG-132 (Supplementary Figure S2B) nor the lysosomal pathway with chloroquine (Supplementary Figure S2C) prevented the drop in hnRNPA1 levels. We therefore further investigated the role of nuclear import as RNPs can be transported into the nucleus via karyopherins. These can be blocked by the peptide inhibitor M9M (19). Expression of this molecule in HEK-293 cells efficiently prevented the cytoplasmic drop in hnRNPA1 levels following 60 min FGF-2 stimulation (Figure 4D). Furthermore, siRNA-mediated silencing of the individual karyopherins, transportin 1 or 2 (TNPO1/TNPO2), demonstrated that downregulation of either protein prevented the decrease in hnRNPA1 cytoplasmic levels (Figure 4E). Consequently, the drop in hnRNPA1 cytoplasmic levels following long-term stimulation with FGF-2 likely results from active import of this ribonuclear-protein (RNP) into the nucleus.

Given our findings above, we considered the possibility that nuclear export of hnRNPA1 might account for the early increase in cytoplasmic levels of this protein after FGF-2 stimulation. To gain a handle on proteins that might be involved, we performed a TAP-tag experiment using hnRNPA1 as the bait in the absence and presence of FGF-2 for 10 min. A full list of the identified interactors and their overlap with the S6K2 TAP results is shown in Supplementary Table S4 and Supplementary Figure S1B/C, respectively. Most of the common interactors were associated with Gene Ontology (GO) terms linked to protein translation and RNA processing (Supplementary Figure S1C). Intriguingly, the hnRNPA1 TAP revealed several mRNA export factors including NXF1 and ALY as interactors (Supplementary Table S4). Since hnRNPA1 was previously shown to interact with mRNAs in the nucleus (12) we hypothesized that association of NXF1 and ALY with hnRNPA1 might be involved in mRNA export. To evaluate this, we first tested whether NXF1 and ALY could indeed associate with hnRNPA1 in an FGF-2-dependent manner. Co-immunoprecipitation experiments in HEK293 cells confirmed that FGF-2 stimulation promoted the interaction of NXF1 with hnRNPA1 (Figure 4F) within a time frame consistent with its increase in the cytoplasmic fraction (Figure 4A). In contrast, ALY was constitutively bound to hnRNPA1 and this interaction remained insensitive to FGF-2 stimulation (Figure 4F). Moreover, silencing of NXF1 using siRNAs prevented the initial peak in hnRNPA1 cytoplasmic levels following FGF-2 treatment (Figure 4G). Thus, the changes in the cytoplasmic levels for hnRNPA1 in FGF-2-treated cells may be explained by a sequential increase in export from and subsequent re-import to the nucleus.

### hnRNPA1 mediates the nuclear export of BCL-XL and XIAP mRNAs in response to FGF-2

**Figure 6.**
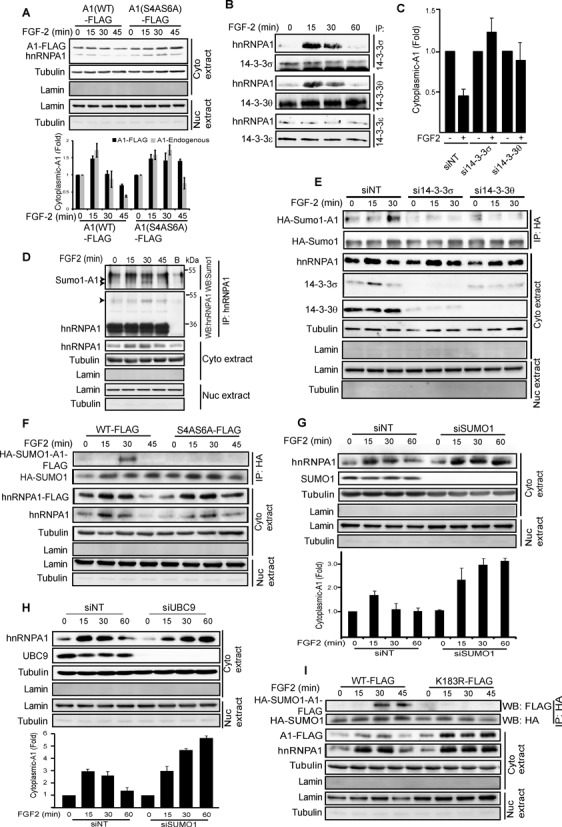
Cytoplasmic hnRNPA1 associates with 14-3-3 subunits and is sumoylated upon stimulation with FGF-2. (**A**, **C**, **G** and **H**) HEK293 cells were transiently transfected with (A) wild-type (WT) or S4AS6A mutant hnRNPA1-FLAG or (C, G and H) siRNAs against the indicated proteins or a non-targeting control (NT) were treated ± FGF-2 for the indicated time (A and G) or 60 min (C) and the cytoplasmic fraction analysed by SDS-PAGE/western blotting (WB). (A, G and H lower panels and C) Band intensities for hnRNPA1 were quantified and normalized to that for tubulin. Results show average of triplicate experiments ± SEM normalised to the corresponding controls. In Fig6A, lower panel shows quantification for both endogenous and the FLAG-tagged hnRNPA1. (**B**) HEK293 cells were treated with FGF-2 and the endogenous 14-3-3 protein subunits σ, θ or ϵ immunoprecipitated (IP) from the cytoplasmic fraction. Immunoprecipitates were analysed by SDS-PAGE/WB for endogenous hnRNPA1 or 14-3-3 proteins. (**D**) Endogenous hnRNPA1 was immunoprecipitated from the cytoplasmic fraction of HEK293 cells treated ± FGF-2. Immunoprecipitates were analysed by SDS-PAGE/WB with either Sumo1 or hnRNPA1 antibodies. The arrows indicate sumoylated-hnRNPA1. (**E**) HEK293 cells stably expressing HA-Sumo1 were treated with siRNA for 14-3-3σ, θ or NT before stimulation with FGF-2. HA-SUMO1 was immunoprecipitated with anti-HA antibodies from the cytoplasmic fraction and the samples analysed by SDS-PAGE/WB for the indicated proteins. (**F** and **I**) HEK293 cells stably expressing HA-SUMO1 were transiently transfected with FLAG-tagged WT or S4AS6A or K183R mutant hnRNPA1 and treated with FGF-2. Cytoplasmic fractions were immunoprecipitated for HA-SUMO1 and analysed as above. All experiments are representative of three independent experiments. See also Supplementary Figure S3.

Given that hnRNPA1 binds both XIAP and BCL-XL mRNAs, we hypothesized that these messages were being co-exported with this RNP from the nucleus following FGF-2 stimulation. To test this, we performed cell fractionation followed by hnRNPA1 immunoprecipitation and qPCR for associated mRNAs. This revealed that the amount of BCL-XL and XIAP mRNA co-precipitated with hnRNPA1 from the cytoplasmic fraction increased during the first 15 min of FGF-2 stimulation (Figure 5A). Silencing of NXF1 prevented this increase (Figure 5B), highlighting the requirement of this component of the RNA export machinery for this process. Moreover, the increase in mRNA export was selective as cIAP1 and GAPDH mRNAs did not show enhanced export (Figure 5A). We then investigated whether the phosphorylation of hnRNPA1 on Ser4/6 impacts on the export of BCL-XL and XIAP mRNAs and their association with this RNP. HEK293 cells were transfected with the WT or non-phosphorylatable hnRNPA1 constructs. The expressed proteins were then immunoprecipitated and qPCR performed to assess the levels of BCL-XL and XIAP mRNAs. Figure 5C shows that FGF-2 stimulation led to the same increase in cytoplasmic hnRNPA1/mRNA complexes as seen with the endogenous protein (Figure 5A). However, expression of the non-phosphorylatable mutant of hnRNPA1 prevented the increase in cytoplasmic BCL-XL and XIAP mRNAs (Figure 5C). Importantly, this did not appear to be a consequence of a failure to export the mutant hnRNPA1 from the cell nucleus as this protein still demonstrated increased abundance in the cytoplasm upon FGF-2 stimulation (Figure 6A). These data suggest that while phosphorylation of hnRNPA1 on Ser4/6 is not required for the nuclear export of this protein, it appears to be necessary for the binding of the RNP to its mRNA cargo. In agreement with this, the phospho-mimetic hnRNPA1 displayed higher affinity for BCL-XL and XIAP mRNAs *in vitro* as compared to the WT or non-phosphorylatable mutant (Figure 5D). Thus, Ser4/6 phosphorylation of hnRNPA1 appears to promote the export of BCL-XL and XIAP mRNAs out of the nucleus in response to FGF-2, thereby increasing the pool of these mRNAs to be translated. Moreover, the binding of BCL-XL or XIAP mRNA to the S4DS6D hnRNPA1 mutant was increased in the nucleus but reduced in the cytoplasm compared to WT protein (Figure 5E). Hence, further events may occur in the cytoplasm to enable hnRNPA1 dissociation from its mRNA cargo promoting their translation. The results also predict that hnRNPA1 downregulation should reduce mRNA export and lead to decreased BCL-XL and XIAP expression and FGF-2 pro-survival signalling. Indeed, siRNA-mediated silencing of hnRNPA1 reduced BCL-XL and XIAP protein levels (Figure 5F) and impaired the induction of these proteins in response to FGF-2 (Figure 5G). Consequently, the FGF-2-mediated pro-survival effect to cisplatin-induced cell death was prevented, as measured by an increased number of cells in the sub-G1 phase of the cell cycle (Figure 5H). Taken together, our data suggest that FGF-2/S6K2-mediated Ser4/6 phosphorylation of hnRNPA1 promotes its binding to BCL-XL and XIAP mRNAs within the nucleus. Furthermore, once exported outside the nucleus this phosphorylation also appears in some way to de-repress cap-independent translation of these mRNAs, thereby facilitating the pro-survival effects of FGF-2.

### hnRNPA1 re-import into the nucleus is dependent on interaction with 14-3-3σ and θ to promote sumoylation

We next focused on unravelling the mechanisms involved in the re-entry of hnRNPA1 into the nucleus. To address this, we first assessed the role of Ser4/6 phosphorylation in this process. Figure 6A shows that in contrast to its WT counterpart, the non-phosphorylatable hnRNPA1 mutant is capable of exiting the nucleus following FGF-2 stimulation, but subsequently fails to be re-imported into the nucleus. This suggested that phosphorylation of Ser4/6 residues may be involved in the import process. A closer examination of the peptide sequence surrounding these phosphosites (MSKSESP) revealed that it closely matched a 14-3-3 consensus sequence (R (S/Ar) (+/Ar) S (L/E/A/M) P). Interaction of cargo proteins with 14-3-3s has been shown to regulate their nuclear import (35,36), and this requires phosphorylation of the 14-3-3 recognition motif (37). Interestingly, our TAP for hnRNPA1 revealed that the σ, θ and ϵ isoforms of 14-3-3 interacted with this RNP in response to FGF-2 (Supplementary Table S4). We therefore next attempted to confirm these interactions by co-immunoprecipitation studies in HEK293 cells. While FGF-2 triggered an inducible interaction between hnRNPA1 and 14-3-3σ and θ the binding of 14-3-3ϵ was constitutive (Figure 6B). The inducible interaction of hnRNPA1 with 14-3-3σ and θ depended on Ser4/6 phosphorylation as, unlike its WT counterpart, the non-phosphorylatable hnRNPA1 mutant failed to interact with these proteins (Supplementary Figure S3A). We therefore reasoned that 14-3-3σ and θ may be necessary for the nuclear import of hnRNPA1 in response to FGF-2. In agreement with this notion, silencing of either of these 14-3-3 isoforms prevented the drop in cytoplasmic hnRNPA1 seen in the control cells following a 60 min treatment with the growth factor (Figure 6C). 14-3-3s often act as scaffolds promoting further post-translational modifications to facilitate protein shuttling (38,39). In this regard, sumoylation of proteins has been shown to target them for nuclear import (40,41). Interestingly, our hnRNPA1 TAP had revealed interactions with a complete sumoylation machinery, including SUMO activating enzyme 1 (SAE1), SUMO activating enzyme 2 (SAE2/UBA2), SUMO conjugating enzyme (UBC9) and the SUMO Ligase E3 (FUS) in response to FGF-2 stimulation (Supplementary Table S4). Hence, we investigated whether stimulation of HEK293 cells with FGF-2 led to the sumoylation of hnRNPA1. Figure 6D shows that FGF-2 treatment induced the Sumo1 modification of hnRNPA1 in a time frame consistent with prior 14-3-3 interaction (Figure 6B). Accordingly, silencing of 14-3-3σ or θ prevented the sumoylation of hnRNPA1 (Figure 6E). Moreover, the hnRNPA1 S4/6A mutant that fails to bind these 14-3-3s was also not sumoylated in response to FGF-2 (Figure 6F). If sumoylation of hnRNPA1 is required for its nuclear import, then mutating the sumoylation site(s) or silencing the machinery required for this post-translational modification should lead to the cytoplasmic accumulation of hnRNPA1 in response to FGF-2. Figure 6G demonstrates that siRNA-mediated silencing of Sumo1 did not inhibit the exit of hnRNPA1 from the nucleus, but instead prevented the subsequent re-import of this protein following FGF-2 stimulation. Similarly, silencing of the SUMO conjugating enzyme, UBC9, led to cytoplasmic accumulation of hnRNPA1 (Figure 6H). Three sumoylation sites (K3, K8 and K183) were predicted within the hnRNPA1 sequence. To determine which of these were relevant for FGF-2 signalling, each was individually mutated. This showed that mutation of K183 to R alone prevented sumoylation of hnRNPA1 (Figure 6I). Consistent with our findings, following Sumo1 or UBC9 silencing, the non-sumoylatable K183R hnRNPA1 mutant was prevented from re-import to the nucleus following FGF-2 stimulation (Figure 6I), establishing the requirement for these sumoylation sites in the nuclear import of hnRNPA1. In contrast, mutation of the K3 or K8 residues had no effect on hnRNPA1 shuttling (Supplementary Figure S3B).

**Figure 7.**
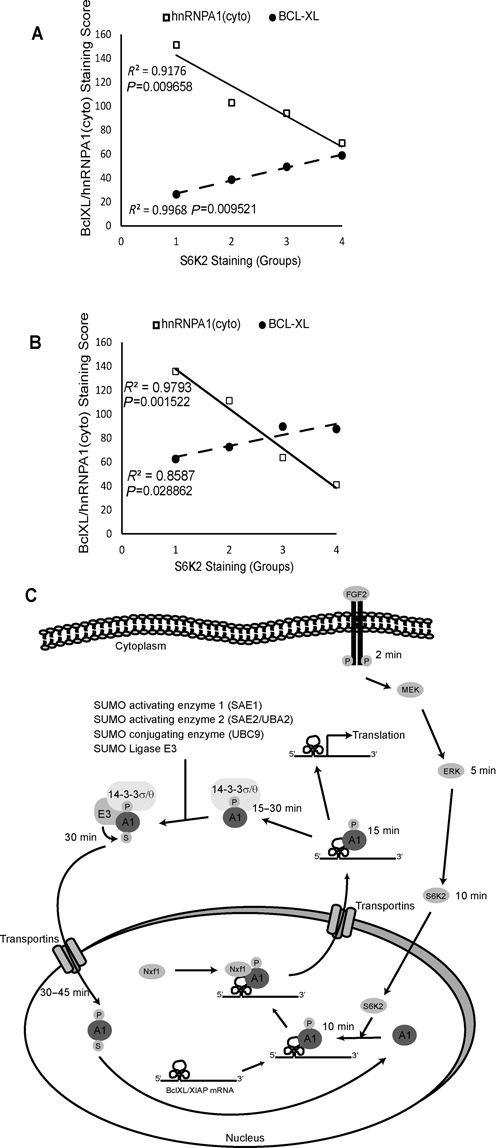
Immunohistochemical staining for S6K2, hnRNPA1 and BCL-XL. TMA samples from lung (*n* = 204) (**A**) and breast cancer (*n* = 194) (**B**) patients were stained for S6K2, hnRNPA1 and BCL-XL. An increase in S6K2 staining correlates with concomitant increase in BCL-XL and decrease in cytoplasmic hnRNPA1 staining. Patients were grouped (X axis; group 1–4) according to their staining scores for S6K2 and correlation curves between the staining intensities for BCL-XL and cytoplasmic hnRNPA1 with groups 1–4 plotted. Correlation coefficients and *P-*values (ANOVA test) are shown. (**C**) Schematic illustration of how FGF-2 signalling regulates the shuttling of hnRNPA1 and the translation of BCL-XL and XIAP mRNAs.

The *in vivo* relevance of our findings on the role of S6K2 signalling to the subcellular localisation of hnRNPA1 is supported by our data from immunohistochemistry staining of lung and breast cancer tissue microarrays. Indeed, the analysis of lung tumours from 204 patients revealed that an increase in S6K2 staining correlates with the concomitant upregulation of BCL-XL (*R*^2^ = 0.9968, *P* = 0.009521) and decrease in cytoplasmic hnRNPA1 staining (*R*^2^ = 0.9176, *P* = 0.009658) (Figure 7A and Supplementary Figure S6A). A similar pattern emerged upon analysis of breast tumours from 194 patients whereby upregulation of BCL-XL (*R*^2^ = 0.8587, *P* = 0.028862) and decrease in cytoplasmic hnRNPA1 (*R*^2^ = 0.9793, *P* = 0.001522) staining correlated with an increase in S6K2 staining (Figure 7B and Supplementary Figure S6B). Taken together, our results demonstrate that FGF-2/S6K2 signalling controls the cycling of hnRNPA1 in and out of the nucleus using stepwise phosphorylation and sumoylation events that drive specific protein–protein interactions (Figure 7C). This cycling is necessary for increased translation of BCL-XL and XIAP and the pro-survival function of FGF-2.

## DISCUSSION

### hnRNPA1 is a substrate of S6K2 and regulates BCL-XL and XIAP translation

We have previously shown that FGF-2/S6K2 signalling enhances the translation of a series of anti-apoptotic proteins including BCL-XL, Bcl-2, XIAP and cIAP1/2 (4–6). This process mediated resistance to cell death induced by several cytotoxic drugs in lung, breast, osteosarcoma and prostate cancer cells ((4,42) and Roy *et al.*, unpublished data). However, these prior studies failed to address the molecular mechanism through which S6K2 could enhance the translation of BCL-XL and XIAP. We previously uncovered PDCD4 as a novel substrate of S6K2 that regulates translation of mRNAs containing IRESs (11). Here, we demonstrate that PDCD4 and S6K2 interact as part of an FGF-2 inducible multi-protein complex that includes the RNA-binding protein hnRNPA1. Our data show that this RNP is indispensable to the pro-survival response downstream of FGF-2/S6K2 signalling as the phosphorylation of hnRNPA1 by S6K2 modulates its binding to the IRESs of BCL-XL and XIAP mRNAs and upregulates their translation.

The involvement of hnRNPA1 in mRNA splicing has been recognised for some time (43–46), but a role in translational regulation has only recently been appreciated (9,10,16,47). In particular, hnRNPA1 has been implicated in the translation of mRNAs containing IRESs in their 5′-UTRs. Such mRNAs are usually poorly translated in normal growing conditions but demonstrate enhanced translation in conditions of cell stress (8,48). In cancer, several oncogenes and tumour promoters are translationally controlled by hnRNPA1, including cMYC, Cyclin D1, FGF-2, XIAP (10,17,49) and BCL-XL (this report), all of which possess IRESs in their mRNAs. Our results reveal the stepwise mechanism by which hnRNPA1 enhances the translation of BCL-XL and XIAP. The first step involves mRNA/hnRNPA1 binding in the nucleus facilitating mRNA export to the cytoplasm. This is followed by the cytoplasmic dissociation of hnRNPA1/mRNA complexes to de-repress IRES-mediated translation prior to the re-import of hnRNPA1 into the nucleus (Figure 7). This process is selective as the export of cIAP1 mRNA was not regulated by hnRNPA1 in response to FGF-2. This suggests that a separate mechanism exists to enhance the translation of cIAP1 downstream of FGF-2/S6K2 signalling which may or may not include hnRNPA1. So how are these steps regulated?

### Two sequential post-translational modifications control hnRNPA1 function

Our results reveal that these steps are controlled by at least two sequential post-translational modifications of hnRNPA1: phosphorylation of Ser4/6 in the N-terminus RNA recognition motif 1 (RRM1) followed by sumoylation of K183 within the RRM2. Both RRMs have been shown to mediate the binding of hnRNPA1 to mRNAs (50). Interestingly, the N-terminus of RRM1 (where Ser4 and 6 are situated) and the C-terminus of RRM2 (where K183 is located) were predicted through modelling to be closely juxtaposed and to be directly involved in RNA binding (51). Indeed, we found that Ser4/6 phosphorylation increases the affinity of hnRNPA1 for the IRESs of BCL-XL and XIAP mRNAs in a cell-free system and their mRNAs in the nucleus of intact cells. Conversely, in the cytoplasm, Ser4/6 phosphorylation targets hnRNPA1 for sumoylation on K183, an event that correlates with the dissociation of this RNP from passenger-mRNAs. Hence, we speculate here that these two post-translational modifications lead to conformational changes in the RRMs that control their mRNA binding ability.

While the phosphorylation of hnRNPA1 on Ser4/6 promotes nuclear export of the bound BCL-XL and XIAP mRNAs, in this state, translation is repressed. However, our data suggest that, in the cytoplasm, the decrease in affinity of hnRNPA1 for its cargo mRNA enables their subsequent translation. The latter correlates with K183 sumoylation which is necessary for re-entry of hnRNPA1 into the nucleus thereby depleting the cytoplasmic pool of this protein. We hypothesize that this depletion could facilitate hnRNPA1 dissociation from and translation of target mRNAs. This notion is supported by the observation that overexpression of an hnRNPA1 mutant that is restricted to the cytoplasm (10), or of the WT protein in the absence of upstream signalling, leads to decreased BCL-XL and XIAP IRES activity and protein expression.

### Possible relevance to cancer and areas for further investigation

At first sight, prior reports demonstrating the translational repression of XIAP and BCL-XL through hnRNPA1 binding would suggest that overexpression of this RNP in cancer could be associated with reduced levels of these anti-apoptotic proteins. This should link to enhanced responsiveness to cytotoxic therapies and improved patient survival. Instead, increased hnRNPA1 expression correlates with worse patient survival (52,53). Our data now provide a mechanism to explain this apparent conundrum. Thus, FGF-2/S6K2 signalling enables post-translational modifications of hnRNPA1 to facilitate both the nuclear export and subsequent translation of BCL-XL and XIAP mRNAs. It is noteworthy that S6K2 activation is dependent on both MEK/ERK and rapamycin-resistant mTOR signalling pathways (21) that are both hyper-activated in >50% of malignancies as a consequence of activating receptor tyrosine kinase, RAS and RAF mutations or phosphatase inhibition (54–60). Therefore, it is tempting to speculate that S6K2 which we have previously reported to be overexpressed in many lung cancers (4) is active in most cases. Our *in vitro* data suggest that the coordinated over-representation of both S6K2 signalling and hnRNPA1 in tumours would be the optimal combination to stimulate IRES-mediated translation. Moreover, these data suggest that increased hnRNPA1 nucleo-cytoplasmic cycling rather than expression alone would be required for this protein to promote drug resistance. The relevance of our findings to cancer is further supported by the fact that similar results to those in HEK293 cells were obtained in small-cell lung cancer cells (Figures 1 and 4A–D) where FGF-2 drives cytotoxic drug-resistance. Moreover, immunohistochemical staining of lung and breast cancer tissue microarray demonstrates that increased expression of S6K2 correlated with increased BCL-XL and decreased cytoplasmic hnRNPA1 levels in tissue samples (Figure 7A and B and Supplementary Figure S6) providing indications of the *in vivo* relevance of findings.

Although we have identified a number of the protein partners involved in the cycling of hnRNPA1 in response to FGF-2 signalling, some steps in this process still warrant further investigation. For instance, while phosphorylation of Ser4/6 is required to promote mRNA nuclear export through increased hnRNPA1/mRNA affinity, it is not necessary for hnRNPA1 to exit the nucleus. Indeed, this latter step still occurs in the absence of S6K2 as well as with the S4A/S6A non-phosphorylatable mutant. This indicates that the RNA binding and the export of hnRNPA1 are dissociated events. It also highlights that a yet unidentified FGF-2-dependent, MEK/ERK-mediated signalling event underlies hnRNPA1 nuclear exit. Published literature has involved the M9 domain of hnRNPA1 in this process (61,62) and further FGF-2-induced post-translational modifications may occur to regulate it. Also, while we have shown the requirement for K183 sumoylation for hnRNPA1 re-import and identified through our TAP-tag purification most of the machinery involved (SAE1, SAE2, UBC9), we have not yet found the E3-ligase responsible for the last step of the SUMO transfer. In this regard, we did co-purify FUS, an E3-ligase involved in Ebp1 sumoylation (63), with hnRNPA1. However, siRNA-mediated silencing of FUS failed to convincingly prevent K183 sumoylation in our experimental set-up. Consequently, the E3-ligase that fulfils this role remains to be determined.

While hnRNPA1 is not druggable per se, our data showing that S6K2 activity mediates chemoresistance in lung cancer and promotes translation of molecules linked to tumorigenesis ((4,11) and present results) suggests that this kinase may be a promising novel therapeutic target for cancer. Pan-S6K inhibitors have been described (64) that target both S6K1 and 2. However, S6K^−/−^/S6K2^−/−^ double knockdown mice are lethal (65) suggesting that the use of pan-S6K drugs may be toxic in the clinic. Hence, further research into S6K2-selective drug development may lead to efficient novel therapeutic strategies for lung and other cancers.

## SUPPLEMENTARY DATA

Supplementary Data are available at NAR Online.

SUPPLEMENTARY DATA
