# Correction to ‘hnRNPA1 couples nuclear export and translation of specific mRNAs downstream of FGF-2/S6K2 signalling’

**DOI:** 10.1093/nar/gkae525

**Published:** 2024-06-14

This is a correction to: Rajat Roy, Danielle Durie, Hui Li, Bing-Qian Liu, John Mark Skehel, Francesco Mauri, Lucia Veronica Cuorvo, Mattia Barbareschi, Lin Guo, Martin Holcik, Michael J. Seckl, Olivier E. Pardo, hnRNPA1 couples nuclear export and translation of specific mRNAs downstream of FGF-2/S6K2 signalling, *Nucleic Acids Research*, Volume 42, Issue 20, 10 November 2014, Pages 12483–12497, https://doi.org/10.1093/nar/gku953

In the original version of this article, the Lamin blot shown in Figure 6A was inadvertently duplicated in Figure 6H.

The authors have provided the original raw data (Supplementary Data), and new Lamin and Tubulin panels for Figure 6H, shown below.


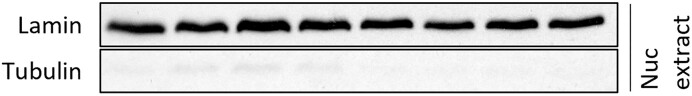


This correction does not affect the results, discussion and conclusions presented in the article. These details have been corrected only in this correction notice to preserve the published version of record.

## Supplementary data

Supplementary Data are available at NAR Online.

## Supplementary Material

gkae525_Supplemental_Files

