# Editor’s Note on “hnRNPA1 couples nuclear export and translation of specific mRNAs downstream of FGF-2/S6K2 signalling”

**DOI:** 10.1093/nar/gkag856

**Published:** 2026-08-26

**Authors:** Julian E Sale, Barry L Stoddard

This is an editor’s note regarding: Rajat Roy, Danielle Durie, Hui Li, Bing-Qian Liu, John Mark Skehel, Francesco Mauri, Lucia Veronica Cuorvo, Mattia Barbareschi, Lin Guo, Martin Holcik, Michael J. Seckl, Olivier E. Pardo, hnRNPA1 couples nuclear export and translation of specific mRNAs downstream of FGF-2/S6K2 signalling, Nucleic Acids Research, Volume 42, Issue 20, 10 November 2014, Pages 12483–12497, https://doi.org/10.1093/nar/gku953

In 2024, the journal published a correction regarding duplication within Figure 6: Correction to ‘hnRNPA1 couples nuclear export and translation of specific mRNAs downstream of FGF-2/S6K2 signalling’, *Nucleic Acids Research*, Volume 52, Issue 13, 22 July 2024, Page 8034, https://doi.org/10.1093/nar/gkae525

In March 2026, the Editors were alerted to concerns about the apparent duplication of blots in additional figures. Specifically:

1. The hnRNPA1 blot in Figure 1C appears identical to the hnRNPA1 blot shown for the 0–15 min FGF2 time course in Figure 4A, despite different experimental conditions.
2. The first four lanes of the Lamin blot in Figure 4B appear highly similar to the nuclear Lamin blot in Figure 5A, despite different experimental conditions.

These new concerns have also been raised on PubPeer: https://pubpeer.com/publications/8E9015B2DE9D9117B2016F1165EFA6#1 and https://pubpeer.com/publications/8E9015B2DE9D9117B2016F1165EFA6#2

The Editors subsequently investigated and contacted the corresponding authors.

**Figure 1C**

The authors acknowledged that the hnRNPA1 panel in the published Figure 1C was incorrectly assembled. They stated that the published hnRNPA1 blot appears to have been inadvertently duplicated from the Figure 4A time-course experiment during figure preparation.

The authors provided:

- a replacement Figure 1C derived from materials retained from their original figure-preparation process; and
- an independent replicate experiment in which the S6K2 and hnRNPA1 immunoprecipitations were analysed on the same blot, together with the associated recovered TIFF image files (**Supplementary File 1**).

The journal’s assessment confirmed that the hnRNPA1 panel in Figure 1C was incorrectly duplicated and does not represent the experiment described in that figure. A corrected figure has therefore been supplied and is published with this Editor’s Note.

**Figure:**
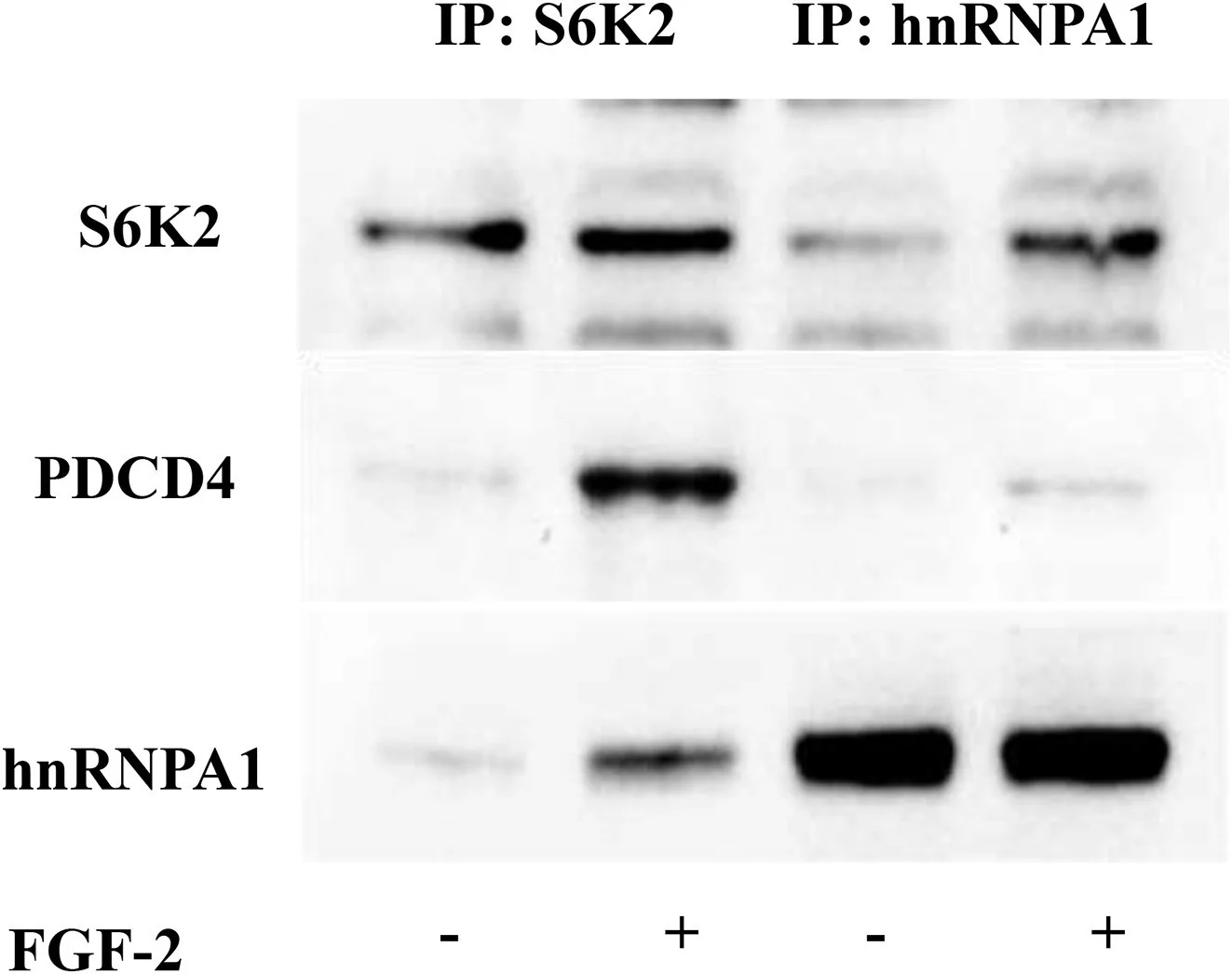


**New Figure 1C**

**Figure 4B and Figure 5A**

The authors disputed that the Lamin blots shown in Figures 4B and 5A are identical. However, they were unable to locate the complete original timestamped blot files for these experiments.

The authors explained that, at the time these experiments were performed, membranes were routinely stained with Ponceau S and cut into smaller sections before antibody incubation. Consequently, archived image files often consisted of individual membrane strips rather than full uncropped membranes. They further stated that cropping to remove empty regions of the exposure area was standard laboratory practice at the time.

The authors provided replacement material corresponding to the experiment, including a revised Figure 4B and higher-resolution images of the associated Lamin blot from the nuclear fraction. The corrected Figure 4B is provided with this notice.

For the concerns regarding Figures 4B and 5A, the original underlying data were not available in sufficient form to enable the journal to determine conclusively whether the apparent similarities between the panels arose from image duplication or another cause. The replacement material supplied by the authors has been made available in the interest of transparency (**Supplementary File 2**).

**Figure:**
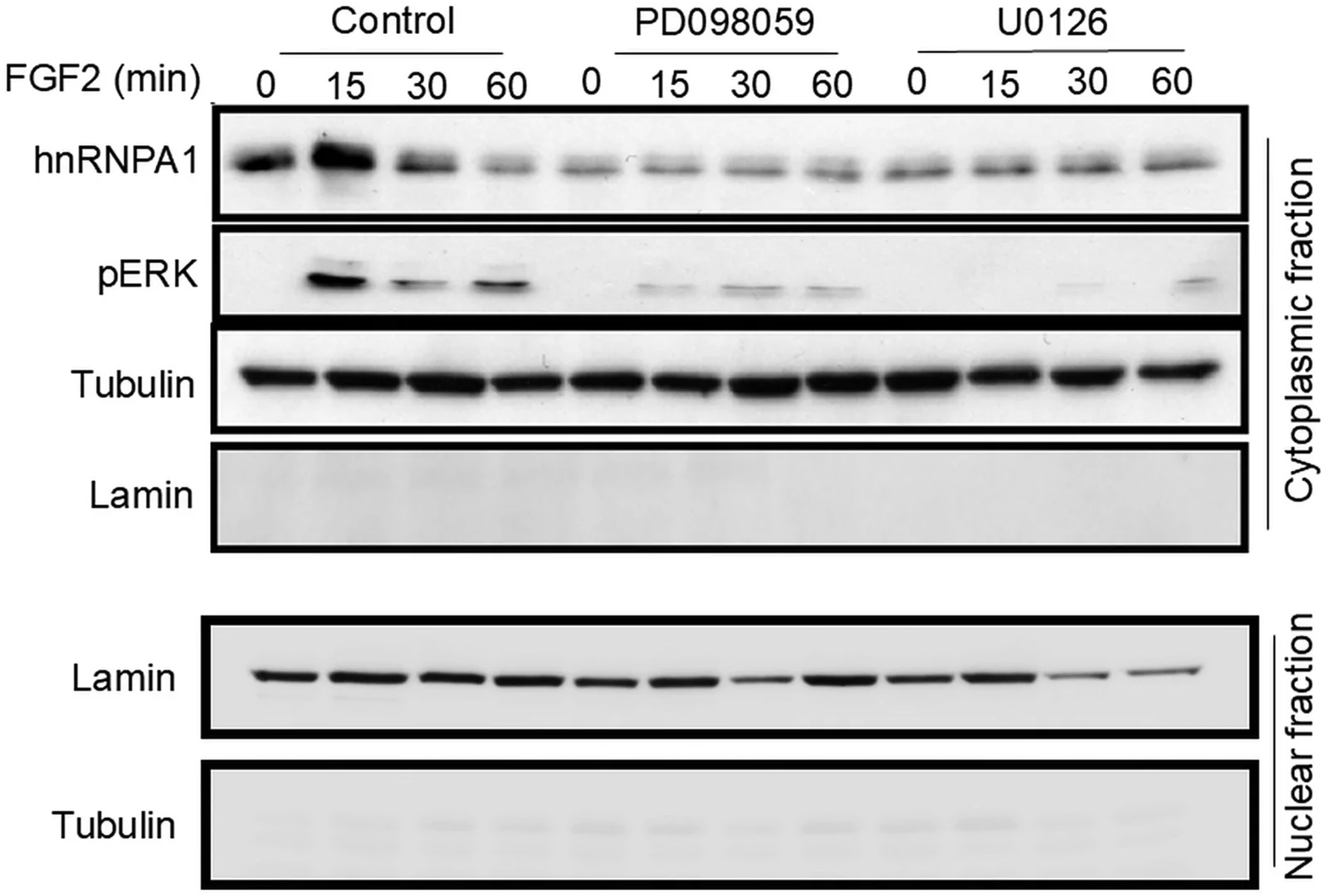


**New Figure 4B**

The journal is publishing this note to document the availability of the underlying source material and the authors’ explanation regarding the preparation of these figures. While these issues may not affect the results, discussion, or conclusions presented in the article, in the absence of original data, the Editors advise readers to examine Figures 1C, 4A, 4B and 5A with care.

## Supplementary Material

gkag856_Supplemental_Files

