# Supplementary material for: hnRNPA1 couples nuclear export and translation of specific mRNAs downstream of FGF-2/S6K2 signalling
Source: Nucleic Acids Res. 2014 Oct 16;42(20):12483–97. doi: 10.1093/nar/gku953 (PMC4227786; doi:10.1093/nar/gku953)
Supplement: SUPPLEMENTARY DATA [file supp_42_20_12483__index.html]

hnRNPA1 couples nuclear export and translation of specific mRNAs downstream of FGF-2/S6K2 signalling — hnRNPA1 couples nuclear export and translation of specific mRNAs downstream of FGF-2/S6K2 signalling — hnRNPA1 couples nuclear export and translation of specific mRNAs downstream of FGF-2/S6K2 signalling — SUPPLEMENTARY DATA 

# hnRNPA1 couples nuclear export and translation of specific mRNAs downstream of FGF-2/S6K2 signalling

## SUPPLEMENTARY DATA

**Files in this Data Supplement:**

- SUPPLEMENTARY DATA
