# Supplementary material for: hnRNPA1 couples nuclear export and translation of specific mRNAs downstream of FGF-2/S6K2 signalling
Source: Nucleic Acids Res. 2014 Oct 16;42(20):12483–97. doi: 10.1093/nar/gku953 (PMC4227786; doi:10.1093/nar/gku953)
Supplement: SUPPLEMENTARY DATA [file supp_gku953_nar-01890-a-2014-File009.pdf]

Supplemental Data

Supplementary Materials and Methods

Reagents and antibodies

Human Recombinant (rec-) FGF-2 and all inhibitors were from CalBiochem; rec-S6K2, Protein-G and -A magnetic beads and Lipofectamine RNAiMAX from Invitrogen; rec-hnRNPA1 from Novus Biological; RNAeasy, Plasmid preparation kits, Attractene, TNPO1 and TNPO2 qPCR quantitect primers from Qiagen; radiolabelled ^{32}P-γ ATP and ^{32}P orthophosphate from Perkin Elmer; Quickchange site-directed mutagenesis kit and RNase inhibitor from Promega. All other reagents were from Sigma Aldrich. Mouse monoclonal antibodies: FLAG (M2), β-actin, α-tubulin, SUMO1 (GMP1), Nxf1, ALY were from Sigma Aldrich; Rabbit polyclonals for BclXL, PDCD4, SUMO1, pERK, UBC9 from Cell Signalling Technologies. Rabbit polyclonal antibody for 14-3-3ε, θ and σ and mouse monoclonals for hnRNPA1 and S6K1 from Santa Cruz. All siRNAs were from Dharmacon.

RNA immunoprecipitation (RNA-IP) qPCR primers

Primers used for qPCR were as follow:

BCL-XL-F: TGCGTGGAAAGCGTAGACAAGGA

BCL-XL-R: AAGGCTCTAGGTGGTCATTCAGGT;

XIAP-F: CCGTGCGGTGCTTTAGTTGT;

XIAP-R: TTCCTCGGGTATATGGTGTCTGAT;

cIAP1-F: GCCTGATGCTGGATAACTGG;

cIAP1-R: GGCGACAGAAAAGTCAATGG;

GAPDH-F: AATCCCATCACCATCTTCCA;

GAPDH-R: TGGACTCCACGACGTACTCA.

Synthetic RNA preparation T7 primers

T7 forward and reverse primers for BCL-XL and XIAP were: BCL-XL–F, TAATACGACTCACTATAGGGCGACAGTAGGAGGCGGAGAGC; BCL-XL-R, TTTTATAATAGAGATGGGCTC;

XIAP-F, TAATACGACTCACTATAGGGCGAAATTAGAATGTTTCTTAGCGGTC; XIAP-R, CTTCTCTTGAAAATAGGAC.

Immunohistochemistry

Tissue microarray (TMA) samples from Lung Cancer patients (n=204) or Breast Cancer patients (n=194) were stained for S6K2, hnRNPA1 and BCL-XL as described earlier (1). A semi quantitative immunohistochemical score (IHS) was used as previously described (2), assessing both the percentage of positive cells and the intensity of staining. For the intensity, a score of 0 to 3, corresponding to negative, weak, moderate and strong positivity, was recorded. The range of possible scores was thus 0 to 300. Two observers blinded to all the clinical data scored all cases and results were 99% consistent. In the 1% of discrepant results a further review was performed by both observers to achieve agreement. Lung tissue samples were further subdivided into Groups 1-4 for increasing S6K2 staining intensity scores, with Group 1 corresponding to negative staining for S6K2 (n=80); Group 2 for intensity of 1-50 (n=60); Group 3 for intensity of 51-70 (n=30) and Group 4 for intensity higher than 70 (n=34). Breast tissue samples were also subdivided into Groups 1-4 for increasing S6K2 staining scores, with Group 1 for intensity 0-80 (n=45); Group 2 for intensity of 81-150 (n=44); Group 3 for intensity of 150-200 (n=38) and Group 4 for intensity of 201-300 (n=66). Average score for BCL-XL and cytoplasmic hnRNPA1 staining intensities for the corresponding groups were calculated and correlation coefficients obtained for S6K2 vs BclXL and S6K2 vs cyto-hnRNPA1 scores. p-values were calculated using ANOVA.

Supplemental References

1. Lara R, Mauri FA, Taylor H, Derua R, Shia A, Gray C, Nicols A, Shiner RJ, Schofield E, Bates PA, Waelkens E, Dallman M, Lamb J, Zicha D, Downward J, Seckl MJ, Pardo OE (2011) An siRNA screen identifies RSK1 as a key modulator of lung cancer metastasis. *Oncogene*

2. Herberger B, Puhalla H, Lehnert M, Wrba F, Novak S, Brandstetter A, Gruenberger B, Gruenberger T, Pirker R, Filipits M (2007) Activated mammalian target of rapamycin is an adverse prognostic factor in patients with biliary tract adenocarcinoma. *Clin Cancer Res* **13:** 4795-4799

Supplementary Table and Figure Legends

Supplementary Tables

Table S1: Quantitative phosphoproteomics analysis of H510 SCLC cells treated with or without FGF-2. Trypsin digests of protein extracts from H510 cells treated with or without FGF-2 were differentially dimethyl-labelled using heavy and light versions of formaldehyde, respectively. Equal amounts of peptides from both conditions were mixed, the phospho-enriched fraction analysed by LC-MS/MS and the light to heavy ratio (L/H) calculated. The experiment was performed three times so as to obtain standard errors (Std. Err.) on the ratio. The gene identifier (ID), sequence of the detected peptide and nature of the modification are indicated. The grayed boxes indicate peptides specifically modified downstream of FGF-2, rather than SCF (Suppl. Fig 3), signalling. (n); position of the modified aa, p; phosphorylation, o; oxidation.

Table S2: List of proteins co-purified in the S6K2-TAP. Tandem affinity purification was performed in HEK293 cells, treated with or without FGF-2, using S6K2 as bait. Tryptic digests were analysed by LC-MS/MS and peptide count for the identified proteins (ID) performed. The difference (Diff) between the peptide count in the absence and the presence of FGF-2 is indicated (<0; decrease, >0; increase, 0; no change following treatment with FGF-2).

Table S3: Quantitative phosphoproteomics analysis of H510 SCLC cells treated with or without SCF. Trypsin digests of protein extracts from H510 cells treated with or without SCF were dimethyl-labelled using heavy and light versions of formaldehyde, respectively. Equal amounts of peptides

from both conditions were mixed, the phospho-enriched fraction analysed by LC-MS/MS and the light to heavy ratio (L/H) calculated. The experiment was performed three times so as to obtain standard errors (Std. Err.) on the ratio. The gene identifier (ID), sequence of the detected peptide and the nature of the modification are indicated. The grayed boxes indicate peptides specifically modified downstream of SCF, rather than FGF-2 (Suppl. Fig 1), signaling. (n); position of the modified aa, p; phosphorylation, o; oxidation.

Table S4: List of proteins co-purified in the hnRNPA1-TAP. Tandem affinity purification was performed in HEK293 cells, treated with or without FGF-2, using hnRNPA1 as bait. Tryptic digests were analysed by LC-MS/MS and peptide count for the identified proteins (ID) performed. The difference (Diff) between the peptide count in the absence and the presence of FGF-2 is indicated (<0; decrease, >0; increase, 0; no change following treatment with FGF-2).

Supplementary Figures

Figure S1: Overlap between phosphoproteomics and TAP-Tag hits. (A) Venn diagram showing the overlap in proteins identified between the phosphoproteomic analysis ± FGF-2 (Suppl. Fig 1) and the S6K2 TAP-Tag (see Suppl. Fig 2). The IDs of the 9 overlapping identified proteins are listed. (B) Venn diagram showing the overlap in proteins identified by TAP to interact with S6K2 and hnRNPA1 (Suppl. Fig 2 and 4). The IDs of the 74 overlapping identified proteins are listed. (C) Gene Ontology (GO) analysis for these 74 proteins listed in (B) shows an enrichment in GO terms associated with translation-related and RNA processing activities.

Figure S2: Cytoplasmic depletion of hnRNPA1 is independent of protein degradation. (A) HEK293 cells were treated with FGF2 for indicated time and subjected to subcellular fractionation. Nuclear and cytoplasmic fractions were separated on SDS-PAGE and probed for the indicated proteins. Detection of Lamin and Tubulin were used to assess the purity of both fractions. HEK293 cells were treated for 1 h with 10μM MG132 (B) or 3 μM Chloroquine (C), concentrations previously shown to inhibit proteosomal degradation and autophagy in our cell system, respectively. The cells were then

stimulated with FGF2 for the indicated time. The cytoplasmic fraction was analysed by SDS-PAGE/Western blotting for the indicated proteins.

Figure S3: FGF2-mediated phosphorylation of hnRNPA1 regulates its interaction with 14-3-3σ and 14-3-3θ leading to hnRNPA1 K183 sumoylation and cytoplasmic depletion. (A) HEK293 cells expressing FLAG-tagged wild type (WT) or phospho-mutant (S4AS6A) hnRNPA1 were stimulated with FGF2 and subjected to immunoprecipitation with 14-3-3 antibodies. Immunoprecipitates were analysed by SDS-PAGE/Western blotting for the indicated proteins. (B) HEK293 cells expressing FLAG-tagged WT or sumoylation mutant (K3R, K8R or K183R) hnRNPA1 were stimulated with FGF2 for the indicated time and the cytoplasmic fraction analysed by SDS-PAGE/Western blotting for the indicated proteins. (C) Multiple sequence alignment for hnRNPA1 sequences from various species. The position of the conserved K183 residue is indicated with an asterisk.

Figure S4: FGF2-dependent modulation of hnRNPA1 in H510 cells. (A) H510 cells were treated $\pm$ FGF2 and the cytoplasmic fraction analysed by SDS-PAGE/Western blotting (WB) for the indicated proteins. (B and C) H510 cells were stimulated $\pm$ FGF2 and the whole cell lysate (B) or the cytoplasmic fraction (C) subjected to immunoprecipitation with hnRNPA1 or 14-3-3 antibodies, respectively. Immunoprecipitates and whole cell lysates were analysed by SDS-PAGE/WB for the indicated proteins. (D) H510 cells were stimulated $\pm$ FGF2 for 30 min and the cytoplasmic fraction subjected to RNA-immunoprecipitation with an hnRNPA1 antibody. hnRNPA1-bound mRNAs were analysed by qPCR.

Figure S5: Schematic of the hnRNPA1 peptide array. 14 amino acid-long overlapping peptides covering the entire sequence of the hnRNPA1 protein were synthesised and covalently coupled to nitrocellulose membranes in 5 rows each containing 30 peptides (Top panel). In the last row, an S6 peptide was spotted in separate 4 positions. The sequence for each peptide and its relative position and molecular weight are indicated in the bottom panel.

Figure S6: Immunohistochemical staining for S6K2, hnRNPA1 and BCL-XL of tissue micro-array samples from lung (A) and breast cancer (B) patients. Sample images for S6K2, BCL-XL and hnRNPA1 staining for a representative patient from each S6K2 score group (upper panel) and the corresponding staining scores (lower panel). Staining for our proteins of interest was revealed using an HRP-conjugated secondary antibody (brown colour). Blue colour; haematoxylin staining. Magnification; x200.

Figure S7: Controls for RNA-IP. (A, C and E): Indicated cells stimulated with or without FGF2 were subjected to immunoprecipitation with hnRNPA1 or FLAG antibodies prior to isolation of the bound BclXL and XIAP mRNA. Half of the immunoprecipates were used for RNA-IP (presented in Figure 5A-5C) and the rest subjected to SDS-PAGE/ Western blotting for the indicated proteins. (B, D and F): Levels of BCLXL and XIAP input mRNA normalized to unstimulated control for the RNA-IP depicted in Figs 5A-C.

Table S1

IDs	z	Sequence	L/H	Std.Err.	Modifications
ABCF1	3	LSVPTSDEEDEVPAPKPR	0.4625	0.1436	(5) p (ST)
ALDOA	3	GILAADESTGSIAKR	0.1519	0.4551	(11) p (ST)
ALG3	2	SGSAAQAEGLCK	0.7455	0.1439	(1) p (ST)
ALG3	2	SGSAAQAEGLCK	0.7455	0.1439	(3) p (ST)
APOOL	2	LGSSSEIEVPAK	1.019	0.05508	(3) p (ST)
BCLAF1	2	IDISPSTLR	0.8161	0.02426	(4) p (ST)
BCLAF1	2	RIDISPSTLR	0.7131	0.1141	(5) p (ST)
BCLAF1	3	DLFDYSPPLHK	0.7851	0.03953	(6) p (ST)
BCLAF1	3	AEGEPQEESPLKSK	1.1	0.07141	(9) p (ST)
BCLAF1	3	LKDLFDYSPPLHK	0.8223	0.02818	(8) p (ST)
BCLAF1	4	LKDLFDYSPPLHK	0.7988	0.03209	(8) p (ST)
BCLAF1	3	KAEGEPQEESPLKSK	0.9994	0.05002	(10) p (ST)
CANX	4	QKSDAEEDGGTVSQEEEDRKPK	1.2	0.03619	(3) p (ST)
CANX	5	QKSDAEEDGGTVSQEEEDRKPK	1.011	0.07812	(3) p (ST)
CANX	4	QKSDAEEDGGTVSQEEEDRKPK	1.2	0.03619	(11) p (ST)
CANX	5	QKSDAEEDGGTVSQEEEDRKPK	1.083	0.04862	(13) p (ST)
CANX	4	QKSDAEEDGGTVSQEEEDRKPK	1.066	0.03205	(3/11) p (ST)
CANX	2	TVIIEQSWGSPK	1.119	0.05165	(10) p (ST)
HSPD1	3	GVMLAVDAVIAELKK	0.6389	0.2678	(3) o (M)
HSPD1	3	ALMLQGVDLLADAVAVTMGPK	0.8979	0.03396	(18) o (M)
HSPD1	3	ALMLQGVDLLADAVAVTMGPK	0.5012	0.3136	(3/18) o (M)
CLDN11	3	FYYTAGSSSPTHAK	1.033	0.05342	(9) p (ST)
CSTF3	4	RPNEDSDEDEEKGAVVPPVHDIYR	0.2414	0.1873	(6) p (ST)
CTNND1	3	GSLASLDSLRK	0.7902	0.1392	(8) p (ST)
DKC1	3	AGLESGAEPGDGDSDTTKK	0.9111	0.226	(14) p (ST)
DLGAP4	3	ANSWQLVETPEKR	0.8508	0.1097	(9) p (ST)
DNAJC5	3	SLSTSGESLYHVLGLDK	1.335	0.02063	(3) p (ST)
DNAJC5	4	SLSTSGESLYHVLGLDKNATSDDIKK	1.357	0.01787	(3) p (ST)
EEF1D	3	ATAPQTQHVSPMR	0.5439	0.02671	(10) p (ST)
ELAVL1	3	NVALLSQLYHSPARR	0.6549	0.1455	(11) p (ST)
FAM134A	2	QALDSEEEEEDVAAK	0.6561	0.1166	(5) p (ST)
FAM54B	2	NASVPNLR	0.5272	0.0653	(3) p (ST)
FAM54B	2	ASSFADMMGILK	0.572	999	(3) p (ST)
FARP1	2	LGAPENSGISTLER	0.5955	0.0461	(11) p (ST)
G3BP1	3	SSSPAPADIAQTVQEDLR	0.4949	0.03259	(1) p (ST)
GFPT1	2	VDSTTCLFPVEEK	0.5651	0.0314	(3) p (ST)
GSK3A	2	GEPNVSYICSR	0.4842	0.04359	(7) p (Y)
HIST1H3E	4	FQSSAVMALQEACEAYLVGLFEDTNLCAIHAK	0.7377	0.02861	(7) o (M)
HIST2H3C	4	FQSSAVMALQEASEAYLVGLFEDTNLCAIHAK	0.7973	0.04751	(7) o (M)
HIST2H3C	5	FQSSAVMALQEASEAYLVGLFEDTNLCAIHAK	0.7564	0.08265	(7/16) o (M)/p (Y)
HIST1H4A	2	RISGLIYEETR	0.7364	0.09754	(3) p (ST)
HDGF	3	AGDLLEDSPKRPK	0.5801	0.06798	(8) p (ST)
HDGF	4	RAGDLLEDSPKRPK	0.5483	0.03258	(9) p (ST)
HMGA1	3	KLEKEEEEGISQESSEEEQ	0.9305	0.0564	(14) p (ST)
HMGA1	4	KQPPVSPGTALVGSQKEPSEVPTPK	0.7742	0.02671	(14) p (ST)
HNRNPH1	3	HTGPNSPDTANDGFVR	0.5924	0.135	(6) p (ST)
HNRNPUL2	2	SGDETPGSEVPGDK	1.15	0.07674	(1) p (ST)
→ HNRNPA1	4	SESPKEPEQLR	1.071	0.02814	(4) p (ST)
→ HNRNPA1	4	SESPKEPEQLR	0.4271	0.0245	(4/6) p (ST)
HNRNPA3	2	SSGSPYGGGYGSGGGSGGYGSR	0.8771	0.01947	(4) p (ST)
HNRNPD	3	FGEVVDCTLKLDPITGR	0.8577	0.03011	(8) p (ST)
HNRNPK	4	IIPTLEEGLQLPSPTATSQLPLESDAVECLNYQHYK	0.6288	0.03428	(15) p (ST)
HNRNPK	4	IIPTLEEGLQLPSPTATSQLPLESDAVECLNYQHYK	0.6288	0.03428	(13) p (ST)
HNRNPU	4	AKSPQPPVEEEDEHFDDTVVCLDTYNCDLHFK	0.9357	0.02836	(3) p (ST)
HSP90AB1	3	IEDVGSDEEDDSGKDK	0.4445	0.07292	(6) p (ST)
HSP90AB1	3	IEDVGSDEEDDSGKDKK	0.5584	0.1461	(6) p (ST)
HUWE1	2	GSGTASDDEFENLR	0.08135	1.567	(6) p (ST)
IRF2BP2	3	RKPSPEPEGEVGPPK	0.5616	0.1568	(4) p (ST)
EIF2B1	2	QGSPVAAGAPAK	0.6052	0.116	(3) p (ST)
EIF5B	3	NKPGPNIESGNEDDDASFK	0.4128	0.07754	(9) p (ST)
ITPR3	2	VASFSIPGSSSR	1.165	0.2199	(3) p (ST)
PRKAR1A	3	TDSREDEISPPPPNPVVK	0.4926	0.01924	(9) p (ST)
PRKACA	3	TWTLCGTPEYLAPEIILSK	0.8217	0.08344	(3) p (ST)
PRKACA	3	GRTWTLCGTPEYLAPEIILSK	0.4866	0.6972	(5) p (ST)
KHDRBS1	3	SGSMDPSGAHPSVR	0.5612	0.09778	(3) p (ST)
KLC2	2	ASSLNFLNK	0.4724	0.06266	(3) p (ST)
KRI1	3	AFVEDSEDEDGAGEGGSSLLQK	0.8696	0.06729	(6) p (ST)
SSB	4	FASDDEHDEHDENGATGPVKR	0.5605	0.05901	(3) p (ST)
TMPO	2	SSTPLPTISSSAENTR	1.35	0.02838	(3) p (ST)
TMPO	4	GPPDFSSDEEREPTPVLGSGAAAAGR	1.056	0.02981	(7) p (ST)
TMPO	2	SSTPLPTISSSAENTR	1.35	0.02838	(3) p (ST)
TMPO	4	GPPDFSSDEEREPTPVLGSGAAAAGR	1.056	0.02981	(7) p (ST)
LMNB2	3	LKLSPSPSSR	1.09	0.3453	(4) p (ST)
MAP1B	2	SDISPLTPR	0.338	0.3653	(7) p (ST)
MAP1B	2	GAESPFEEK	4.78	4.808	(4) p (ST)
MAP1B	2	SPSLSPSPPSPLEK	0.5654	0.1312	(10) p (ST)
MAP1B	3	EVPSKEEPSPVKAEVAEK	1.425	0.04416	(9) p (ST)
MAP1B	3	TATCHSSSSPPIDAASAEPYGFR	0.5801	0.01544	(9) p (ST)
MAP1B	3	TATCHSSSSPPIDAASAEPYGFR	0.5801	0.01544	(7) p (ST)
MAP1B	4	KLGDVSPTQIDVSQFGSFKEDTK	0.6369	0.06581	(8) p (ST)
MAP1B	4	VLSPLRSPPLIGSESAYESFLSADDKASGR	0.4015	0.08954	(7) p (ST)
MAP1B	4	TELSPSFINPNPLEWFASEEPTEESEKPLTQSGGAPPPPGGK	0.5911	0.03628	(6) p (ST)
MATR3	3	SYSPDGKESPSDKK	1.089	0.03185	(3) p (ST)
MATR3	4	RDSFDDRGPSLNPVLDYDHGSR	0.6921	0.01329	(3) p (ST)
BTAF1	4	KESKEEETSIDVAGKPNEVTK	1.218	0.07594	(3) p (ST)
NDRG1	2	TASGSSVTSLDGTR	0.4198	0.02482	(3) p (ST)
NIPBL	2	AITSLLGGGSPK	0.8254	0.05915	(10) p (ST)
NOLC1	3	AESSNSSSSDDSSEEEEEKLK	0.6278	0.06473	(4) p (ST)
NOLC1	3	AASSSSSSSSSSSSDDSEEEKAAATPK	0.9343	0.06137	(6) p (ST)
NOLC1	4	KASSSDSEDSSEEEEEVQGPPAKK	1.079	0.04882	(5) p (ST)
NOLC1	3	AASSSSSSSSSSSSDDSEEEKAAATPK	0.9343	0.06137	(4) p (ST)
NOLC1	4	ASSSDSEDSSEEEEEVQGPPAKK	0.07866	0.1973	(3/4/6) p (ST)
NOLC1	4	KASSSDSEDSSEEEEEVQGPPAKK	0.2931	0.1632	(3/4/5) p (ST)
NOLC1	3	AAESSSDSSDSDSSEDDEAPSKPAGTTK	0.9249	0.1385	(11) p (ST)
NOLC1	4	AAESSSDSSDSDSSEDDEAPSKPAGTTK	0.9953	999	(11) p (ST)
NOLC1	4	ASESSSSEESSDDDDEEDQKKQPVQK	0.988	0.02918	(10) p (ST)
NOLC1	4	QQPVESSEDSSDESDSSSEEEKKPPTK	1.326	0.04023	(18) p (ST)
NOLC1	4	QQPVESSEDSSDESDSSSEEEKKPPTK	1.326	0.04023	(17) p (ST)
NPM1	4	CGSGPVHISGQHLVAVEEDAESEDEEEEDVK	0.9123	0.01619	(22) p (ST)
NUMA1	3	KNSLISSLEEEVSILNR	0.9792	0.3131	(6) p (ST)
NUP35	2	TLGTPTQPGSTPR	0.8788	0.0806	(4) p (ST)
NUP35	3	CALSSPSLAFTPPIK	0.9135	0.1916	(5) p (ST)
NUP35	3	ASTSDYQVISDRQTPK	1.093	0.0591	(10) p (ST)
PDHA1	3	YHGHSMSDPGVSYR	13.18	0.9528	(5) p (ST)
PI4K2A	2	SSSESYTQSFQSR	0.8066	0.05918	(3) p (ST)
PDCD4	2	SGLTVPTSPK	0.4667	0.05456	(8) p (ST)
P4HB	4	FLESGGQDGAGDDDDLEDLEEAEEPDMEEDDDQK	0.563	0.06642	(27) o (M)
PELP1	3	GSPDGSLQTGKPSAPK	1.065	0.07926	(2) p (ST)
PELP1	3	AGSNEDPILAPSGTPPPTIPPDETFGGR	0.8934	0.03656	(14) p (ST)
PGK1	3	ALESPERPFLAILGGAK	0.521	0.05108	(4) p (ST)
PGM2L1	3	AVAGVMITASHNR	0.4828	0.3025	(10) p (ST)
PGRMC1	3	GDQPAASGDSDDDEPPPLPR	1.011	0.06943	(10) p (ST)
PGRMC1	3	EGEEPTVYSDEEEPKDESAR	1.135	0.01816	(9) p (ST)
PGRMC1	3	IVRGDQPAASGDSDDDEPPPLPR	0.8744	0.0497	(13) p (ST)
PGRMC1	4	IVRGDQPAASGDSDDDEPPPLPR	0.7179	0.03222	(13) p (ST)
PGRMC1	4	EGEEPTVYSDEEEPKDESARK	0.9342	0.04012	(9) p (ST)
PGRMC2	3	LLKPGEEPSEYTDEEDTK	5.28	0.8286	(12) p (ST)
PGRMC2	4	LLKPGEEPSEYTDEEDTKDHNKQD	1.221	0.01793	(12) p (ST)
PGRMC2	5	LLKPGEEPSEYTDEEDTKDHNKQD	1.167	0.01493	(12) p (ST)
PHF2	3	ESASPTIPNLDLLEAHTK	0.6593	0.09732	(2) p (ST)
PHF2	3	ESASPTIPNLDLLEAHTK	0.6593	0.09732	(6) p (ST)
NUP210	2	DLAVPAALTPR	0.8498	0.01846	(9) p (ST)
PRRC2A	2	LIPGPLSPVAR	0.6095	0.04117	(7) p (ST)
PRKRA	2	EDSGTFSLGK	0.9999	0.04069	(3) p (ST)
PRKRA	3	AEAPPLEREDSGTFSLGK	0.4907	0.233	(11) p (ST)
PSIP1	2	QSNASSDVEVEEK	1.184	0.1879	(6) p (ST)
PSIP1	4	TGVTSTSDSEEEGDDQEGEKKR	0.6219	0.1233	(7) p (ST)
PUM2	2	QASPTEVVER	0.5308	0.1163	(3) p (ST)
RBM15	3	DRTPPLLYR	0.7448	0.01463	(3) p (ST)
RBM15	2	SLSPGGAALGYR	0.7706	0.02223	(3) p (ST)
EBAG9	3	KLSGDQITLPTTVDYSSVPK	0.8441	0.02869	(3) p (ST)
RCC1	3	RRSPPADAIPK	0.8511	0.01599	(3) p (ST)
RPL12	2	IGPLGLSPK	0.9054	0.03457	(7) p (ST)
RPRD1B	4	TFQQIQEEEDDDYPGSYSPQDPSAGPLLTEELIK	0.4893	0.05211	(18) p (ST)
RPS3	2	DEILPTTPISEQK	1.348	0.07858	(7) p (ST)
RPS3	4	KPLPDHVSIVEPKDEILPTTPISEQK	0.4817	0.151	(20) p (ST)
SLC35B2	2	AVPVESPVQKV	0.9639	0.06393	(6) p (ST)
SLC35B2	2	KAVPVESPVQKV	0.9507	0.07677	(7) p (ST)
SLC35C2	2	GLGSSPDLELLLR	0.3777	0.2572	(4) p (ST)
SMAP	3	SASPDDDLGSSNWEAADLGNEER	0.5969	0.9709	(3) p (ST)
SNIP1	3	QERLSPEVAPPAHR	0.6188	0.02212	(5) p (ST)
SNIP1	3	RPDHSGGSPSPPTSEPAR	43.25	1.397	(8) p (ST)
SRF	3	ALIQTCLNSPDSPPRSDPTTDQR	0.6832	0.04908	(12) p (ST)
SRRM1	2	RYSPSPPPK	0.957	0.2308	(3) p (ST)
SRRM1	3	HRPSPPATPPPK	1.938	0.382	(4) p (ST)
SRRM1	3	HRPSPPATPPPK	0.5813	0.05095	(4/8) p (ST)
SRRM1	3	EKTPELPEPSVK	0.7632	0.02191	(3) p (ST)
SRRM1	3	KVELSESEEDKGGK	0.6744	0.1236	(5) p (ST)
SRRM1	3	KETESEAEDNLDDLEK	0.5255	0.04038	(5) p (ST)
SRRM2	2	GEFSASPMLK	0.6948	0.1041	(6) p (ST)
SRRM2	2	SSSPVTELASR	0.7555	0.06322	(3) p (ST)
SRRM2	2	SCFESSPDPELK	0.5878	0.1016	(6) p (ST)
SRRM2	3	SSTPPGESYFGVSSLQLK	0.7358	0.1477	(3) p (ST)
SRRM2	4	GRGPSPEGSSSTESSPEHPPK	0.7115	0.1034	(15) p (ST)
SRRM2	4	RGEGDAPFSEPGTTSTQRPSSPETATK	0.7413	0.0383	(21) p (ST)
SRRT	3	TQLWASEPGTPPLPTSLPSQNPILK	0.6944	0.03041	(10) p (ST)
SRSF11	3	DYDEEEQGYDSEKEK	0.4614	0.1508	(11) p (ST)
SRSF9	3	GSPHYFSPFRPY	0.8182	0.02475	(2) p (ST)
SSFA2	2	SQSLPTTLLSPVR	0.6505	0.09345	(3) p (ST)
SSRP1	2	EFVSSDESSSGENK	0.9139	0.09501	(9) p (ST)
STMN1	2	ASGQAFELILSPR	0.3397	0.1112	(11) p (ST)
STT3B	3	ENPPVEDSSDEDDKR	1.069	0.07752	(8) p (ST)
STT3B	3	ENPPVEDSSDEDDKR	1.478	0.1932	(8/9) p (ST)
TCOF1	3	TSQVGAASAPAKESPR	0.6248	0.05401	(14) p (ST)
TCOF1	3	LGAGEGGEASVSPEKTSTTSK	0.846	0.06791	(12) p (ST)
SUB1	3	ELVSSSSSGSDSDSEVDKK	0.9933	0.0778	(12) p (ST)
TERF2IP	2	YLLGDAPVSPSSQK	0.4253	0.07066	(9) p (ST)
TMX1	3	KVEEEQEADEEDVSEEEAESK	0.4386	0.04346	(14) p (ST)
TP53BP1	2	STPFIVPSSPTEQEGR	0.4648	0.03843	(9) p (ST)
SPAG1	3	KQSLGELIGTLNAAK	0.5493	0.05823	(3) p (ST)
SPAG1	4	KQSLGELIGTLNAAKVPADTEVVCAPPTAYIDFAR	0.2037	0.08737	(10) p (ST)
THRAP3	2	ASAVSELSPR	0.83	0.01135	(8) p (ST)
THRAP3	2	IDISPSTFR	0.8023	0.04372	(4) p (ST)
THRAP3	2	RIDISPSTFR	0.9014	0.02172	(5) p (ST)
THRAP3	3	ERSPALKSPLQSVVVR	0.6833	0.01584	(3) p (ST)
UBE2J1	3	RLSTSPDVIQGHQPR	0.8016	0.1246	(3) p (ST)
VDAC1	3	LTFDSSFSPNTGKK	0.924	0.04589	(8) p (ST)
VDAC2	3	LTFDTTFSPNTGKK	0.4555	0.1694	(8) p (ST)
KIAA1429	2	SFLSEPSSPGR	1.026	0.08994	(8) p (ST)
ZNF828	2	GGSPDLWK	0.9038	0.0415	(3) p (ST)

Table S2

IDs	Name	Diff
A0A5E5	Putative uncharacterized protein	-1
ACPP	Acid phosphatase 1 isoform c variant	-1
ACTA1	Actin, alpha 1, skeletal muscle	-1
ACTG1	Actin, cytoplasmic 2	-1
AIFM1	Apoptosis-inducing factor, mitochondrion-associated, 1	1
ALB	Albumine	1
ALG1	Chitobiosyldiphosphodolichol beta-mannosyltransferase	-1
ANKMY2	Ankyrin repeat and MYND domain containing 2	0
ANXA2	Annexin A2	-2
ATP1A1	Sodium/potassium-transporting ATPase subunit alpha-1	-4
ATP5A1	ATP synthase subunit alpha, mitochondrial	1
BAG2	BAG family molecular chaperone regulator 2	1
BRAF	v-raf murine sarcoma viral oncogene homolog B1	4
C22orf28	Putative uncharacterized protein HSPC117	-2
CAD	Carbamoyl-phos ∑ase 2, Asp transcarbamylase, and dihydroorotase	0
CAPRIN1	Caprin-1	0
CAPZA1	F-actin-capping protein subunit alpha-1	-1
CCDC124	Coiled-coil domain-containing protein 124	0
CCT2	T-complex protein 1 subunit beta	-3
CCT3	T-complex protein 1 subunit gamma	-3
CCT4	T-complex protein 1, delta subunit	1
CCT5	T-complex protein 1 subunit epsilon	-3
CCT6A	Chaperonin subunit 6A	-1
CCT7	T-complex protein 1 subunit eta	0
CCT8	T-complex protein 1 subunit theta	2
CDC37	Hsp90 co-chaperone Cdc37	1
CDC45	Cell division cycle 45 homolog	-3
CFL1	Cofilin-1	-2
CLNS1A	Methylosome subunit pICln	0
DDB1	DNA damage-binding protein 1	3
DDX1	ATD-dpt RNA helicase DEAD box polypep 1	0
DDX17	ATD-dpt RNA helicase DEAD box polypep 17	1
DDX3X	ATD-dpt RNA helicase DEAD box polypep 3X	2
DDX5	ATD-dpt RNA helicase DEAD box polypep 5	-2
DDX6	ATD-dpt RNA helicase DEAD box polypep 6	1
DHX9	ATD-dpt RNA helicase DEAD box polypep 9	2
DNAJA2	DnaJ (Hsp40) homolog, subfamily A, member 2	1
DNAJB1	DnaJ homolog subfamily B member 1	-1
DNAJC7	DnaJ homolog subfamily C member 7	3
DNJA1	DnaJ homolog subfamily A member 1	1
DNJA2	DnaJ homolog subfamily A member 2	1
EEF1A1	Elongation factor 1-alpha 1	-3
EEF1G	Elongation factor 1-gamma	-1
EIF2S2	Eukaryotic translation initiation factor 2, subu 2 beta	-2
EIF3B	Eukaryotic translation initiation factor 3, subunit 9 eta	1
EWSR1	RNA-binding protein Ewing sarcoma breakpoint region 1	-1
FAM60A	Transitional endoplasmic reticulum ATPase	-1
FKBP5	FK506-binding protein 5	8
FKBP8	FK506-binding protein 8	1
FUS	Fused in sarcoma	1
G3BP1	Ras GTPase-activating protein-binding protein 1	1
GDF5	Growth differentiation factor 5	0
GTF2I	General transcription factor II-I	3
HAT1	Histone acetyltransferase 1	1
hCG2043616	leucine-rich repeat-containing protein 15 isoform	-1
HLA-A	HLA class I alpha chain	0
→ HNRNPA1	Heterogeneous nuclear ribonucleoproteins A1	4
HNRNPA2B1	Heterogeneous nuclear ribonucleoproteins A2/B1	0
HNRNPK	Heterogeneous nuclear ribonucleoprotein K	-1
HNRNPM	Heterogeneous nuclear ribonucleoprotein M	0
HNRNPU	Heterogeneous nuclear ribonucleoprotein U	2
HSP90AA1	Heat shock protein HSP 90-alpha	5
HSP90AB1	Heat shock protein 90kDa alpha class B member 1	6
HSPA14	Heat shock 70kDa protein 14	1
HSPA1A	Heat shock 70 kDa protein 1	4
HSPA4L	Heat shock 70 kDa protein 4-like protein	0
HSPA5	78 kDa glucose-regulated protein	3
HSPA6	Heat shock 70 kDa protein 6	0
HSPA8	Heat shock cognate 71 kDa protein	-4
HSPA9	Stress-70 protein, mitochondrial	3
HSPD1	60 kDa heat shock protein, mitochondrial	1
HSPH1	Heat shock protein 105 kDa	-2
IGF2BP1	Insulin-like growth factor 2 mRNA-binding protein 1	-1
IPO5	Importin 5	0
IPO7	Importin-7	-1
IQGAP1	IQ motif containing GTPase activating protein 1	-2
KIF2A	Kinesin-like protein KIF2A	0
KIF5B	Kinesin-1 heavy chain	1
KRT1	Keratin, type II cytoskeletal 1	-4
KRT10	Keratin, type I cytoskeletal 10	-1
KRT13	Keratin, type I cytoskeletal 13	2
KRT14	Keratin, type I cytoskeletal 14	-5
KRT16	Keratin, type I cytoskeletal 16	-3
KRT2	Keratin, type II cytoskeletal 2 epidermal	0
KRT5	Keratin, type II cytoskeletal 5	-6
KRT6A	Keratin, type II cytoskeletal 6A	-2
KRT9	Keratin, type I cytoskeletal 9	0
LRPPRC	Leucine-rich PPR motif-containing protein, mitochondrial	2
LTV1	LTV1 homolog	-1
LV302	Ig lambda chain V-III region LOI	0
LYZ	Lysozyme C	1
MAGED1	Melanoma-associated antigen D1	-1
MAGED2	Melanoma-associated antigen D2	2
MCM3	Cervical cancer proto-oncogene 5	-3
MRPS22	28S ribosomal protein S22, mitochondrial	-1
MSH6	G/T mismatch-binding protein	0
MYO1C	Myosin-Ic	1
NCL	Nucleolin	3
NME3	Non-metastatic cells 3, protein expressed in	-1
NONO	Non-POU domain-containing octamer-binding protein	-3
NPM1	Nucleolar phosphoprotein B23	1
NUDC	Nuclear distribution gene C homolog	-1
PCBP1	Poly(rC)-binding protein 1	-2
PCMT1	Protein-L-isoaspartate O-methyltransferase	1
PCNA	Proliferating cell nuclear antigen	2
PDCD4	Programmed cell death protein 4	2
PHGDH	Phosphoglycerate dehydrogenase	0
POLDIP3	DNA-directed polymerase , delta interacting protein 3	-1
PPM1G	Protein phosphatase 1G	-3
PPP2CA	S/T-protein phosphatase 2A catal subu alpha isoform	-1
PPP2R1A	S/T-protein phosphatase 2A reg subu A alpha	-2
PPP2R2A	S/T-protein phosphatase 2A reg subu B alpha	2
PPP6R1	Ser/thr-protein phosphatase 6 regulatory subu 1	1
PPP6R3	Ser/thr-protein phosphatase 6 regulatory subu 3	-3
PPYR1	pancreatic polypeptide receptor 1	0
PRDX1	Peroxiredoxin 1	3
PRMT1	Protein arginine N-methyltransferase 1	0
PRSS3	Trypsin-3	1
PSMC1	26S protease regulatory subunit 4	1
PSMC2	Proteasome (Prosome, macropain) 26S subunit, ATPase, 2	-4
PSMC3	26S protease regulatory subunit 6A	-1
PSMD3	proteasome 26S subu, non-ATPase, 3	-1
PSPC1	Paraspeckle component 1	-2
PTGES3	Prostaglandin E synthase 3	0
Q59GD0	Polo-like kinase variant	0
RBBP7	Retinoblastoma binding protein 7	-1
RPL13	60S ribosomal protein L13	1
RPL14	60S ribosomal protein L14	0
RPL17	60S ribosomal protein L17	-1
RPL19	60S ribosomal protein L19	1
RPL22L1	Ribosomal protein L22-like protein	1
RPL23A	60S ribosomal protein L23a	2
RPL24	Ribosomal protein L24	1
RPL26	60S ribosomal protein L26	1
RPL8	60S ribosomal protein L8	2
RPN1	Ribophorin I	-1
RPS14	Ribosomal protein S14	1
RPS17	40S ribosomal protein S17	-2
RPS18	40S ribosomal protein S18	-4
RPS19	40S ribosomal protein S19	-1
RPS2	40S ribosomal protein S2	-1
RPS25	40S ribosomal protein S25	-1
RPS3	Ribosomal protein S3	-4
RPS3A	40S ribosomal protein S3a	-2
RPS4X	40S ribosomal protein S4, X isoform	-1
RPS5	40S ribosomal protein S5	1
RPS6	40S ribosomal protein S6	0
RPS7	Ribosomal protein S7	2
RPS8	40S ribosomal protein S8	1
RUVBL1	RuvB-like 1	0
RUVBL2	RuvB-like 2	2
SERBP1	Plasminogen activator inhibitor 1 RNA-binding protein	1
SFPQ	Splicing factor, proline- and glutamine-rich	6
SHROOM3	Shroom family member 3	1
SLC25A5	ADP/ATP translocase 2	1
SLC3A2	4F2 cell-surface antigen heavy chain	-1
SNRPB	Small nuclear ribonucleoprotein polypeptide B''	-1
SSR1	Translocon-associated protein subu alpha	-1
STUB1	STIP1 homology and U-box containing protein 1	1
SUGT1	Suppressor of G2 allele of SKP1 homolog	-1
TAF15	RNA polymerase II, TBP-associated factor	-3
TCP1	T-complex protein 1 subunit alpha	0
TFAM	Mitochondrial transcription factor A	1
TIMM50	Mitochondrial import inner membrane translocase subu 50	0
TUBA1B	Tubulin alpha-1B chain	-1
TUBA1C	Tubulin alpha-1C chain	-1
TUBB	Tubulin beta chain	-1
TUBB2C	Tubulin beta-2C chain	0
TUBB3	Tubulin beta-3 chain	0
TUBB6	Tubulin beta-6 chain	-1
TUFM	Elongation factor Tu, mitochondrial	0
UBIQ	Ubiquitin	0
USP7	Ubiquitin carboxyl-terminal hydrolase 7	2
USP9X	Ubiquitin specific peptidase 9, X-linked	2
WBSCR22	Williams Beuren syndrome chromosome region 22	-1
WRNIP1	Werner helicase interacting protein 1	2
XRCC5	X-ray repair complementing defective in Chinese hamster cells 5	0
YBX1	Nuclease-sensitive element-binding protein 1	1
YTHDF2	YTH domain family protein 2	-2
YWHAH	14-3-3 protein eta	1
YWHAQ	Tyr 3-/Trp 5-monooxygenase activation prot, theta polypep	-1

Table S3

ID	z	Sequence	L/H	Std.Err.	Modifications
APOOL	2	LGSSSEIEVPAK	0.9716	0.07473	(4) p (ST)
APOOL	2	LGSSSEIEVPAK	0.9716	0.07473	(3) p (ST)
BCLAF1	2	IDISPSTLR	0.9337	0.02592	(4) p (ST)
BCLAF1	3	IDISPSTLRK	0.9943	0.05885	(4) p (ST)
BCLAF1	2	RIDISPSTLR	1.026	0.02483	(5) p (ST)
BCLAF1	3	DLFDYSPPLHK	0.847	0.03403	(6) p (ST)
BCLAF1	2	KAEGEPQEESPLK	0.9641	0.0352	(10) p (ST)
BCLAF1	3	LKDLFDYSPPLHK	0.9087	0.03193	(8) p (ST)
BCLAF1	3	KAEGEPQEESPLKSK	0.9401	0.03384	(10) p (ST)
CANX	4	QKSDAEEDGGTVSQEEEDRKPK	1.017	0.03517	(3) p (ST)
CANX	4	QKSDAEEDGGTVSQEEEDRKPK	0.9109	0.01956	(3/13) p (ST)
CERS2	4	LVEDERSDREETESSEGEEAAAGGGAK	0.7982	0.04767	(7) p (ST)
CERS2	4	LVEDERSDREETESSEGEEAAAGGGAK	0.7982	0.04767	(12) p (ST)
CERS4	3	SDVEESDSSEEAAAAQEPLQLK	0.732	0.05889	(8) p (ST)
CIR1	3	NLTANDPSQEYVASEGEEDPEVEFLK	0.8518	0.03777	(14) p (ST)
COBRA1	3	KPSPAQAAETPALELPLPSVPAPAPL	0.9109	0.01961	(3) p (ST)
CTAGE5	2	AFLSPPTLLEGPLR	0.8464	0.04096	(4) p (ST)
CTNND1	3	GSLASLDSLRK	0.7647	0.1562	(8) p (ST)
DHX9	5	SEEVPAFGVASPPPLTDTPDTTANAEGDLPTTMGGPLPPHLALK	0.8341	0.08473	(11) p (ST)
DKC1	2	AGLESGAEPGDGDSDTTK	0.8505	0.04068	(14) p (ST)
DNAJC5	3	SLSTSGESLYHVLGLDK	1.146	0.02592	(1) p (ST)
DNAJC5	3	SLSTSGESLYHVLGLDK	1.12	0.01924	(3) p (ST)
DNAJC5	4	SLSTSGESLYHVLGLDKNATSDDIKK	0.9176	0.007932	(3) p (ST)
DSG2	3	VVPSFLPVDQGGSLVGR	0.9982	0.06179	(13) p (ST)
DSP	3	SSSFSDTLEESSPIAAIFDTENLEK	0.865	0.04258	(2) p (ST)
DSP	3	SSSFSDTLEESSPIAAIFDTENLEK	0.865	0.04258	(7) p (ST)
DTD1	2	SASSGAEGDVSSEREP	0.7591	0.06806	(4) p (ST)
EBAG9	3	KLSGDQITLPTTVDYSSVPK	0.8605	0.03228	(3) p (ST)
EEF1D	3	ATAPQTQHVSPMR	0.6694	0.02365	(10) p (ST)
EIF2B1	2	QGSPVAAGAPAK	0.9968	0.0263	(3) p (ST)
EIF3G	4	GIPLATGDTSPEPELLPGAPLPPPK	0.9113	0.05062	(10) p (ST)
EIF5B	3	NKPGPNIESGNEDDDASFK	0.8088	0.04501	(9) p (ST)
FAM54B	2	NASVPNLR	0.8438	0.05184	(3) p (ST)
FAM54B	2	ASSFADMMGILK	0.593	0.1001	(3) p (ST)
FARP1	2	LGAPENSGISTLER	0.9069	0.03999	(11) p (ST)
FOXK1	3	EGSPIPHDPEFGSK	0.9948	0.04704	(3) p (ST)
G3BP1	2	SSSPAPADIAQTVQEDLR	0.9217	0.08376	(3) p (ST)
GFPT1	2	VDSTTCLFPVEEK	1	0.04542	(3) p (ST)
GORASP2	3	VGDSTPVSEKPVSAAVDANASESP	0.8271	0.03414	(23) p (ST)
GSK3B	2	GEPNVSYICSR	0.9331	0.04698	(7) p (Y)
HDGF	3	AGDLLEDSPKRPK	0.8422	0.06093	(8) p (ST)
HDGF	4	RAGDLLEDSPKRPK	0.723	0.04047	(9) p (ST)
HMGA1	3	KLEKEEEEGISQESSEEEQ	0.7618	0.05164	(14) p (ST)
HMGA1	4	KQPPVSPGTALVGSQKEPSEVPTPK	0.9048	0.02954	(14) p (ST)
HNRNPA3	2	SSGSPYGGGYGSGGGSGGYGSR	0.9461	0.03465	(4) p (ST)
HNRNPK	5	IIPTLEEGLQLPSPTATSQLPLESDAVECLNYQHYK	0.7945	0.01811	(17) p (ST)
HNRNPK	4	IIPTLEEGLQLPSPTATSQLPLESDAVECLNYQHYK	0.8176	0.0176	(13) p (ST)
HNRNPK	5	KIIPTLEEGLQLPSPTATSQLPLESDAVECLNYQHYK	0.7708	0.05395	(19) p (ST)
HNRNPU	5	AKSPQPPVEEEDEHFDDTVVCLDTYNCDLHFK	0.7828	0.01885	(3) p (ST)
HNRNPU	5	AKSPQPPVEEEDEHFDDTVVCLDTYNCDLHFK	0.7828	0.01885	(18) p (ST)
HNRNPUL2	2	SGDETPGSEVPGDK	0.9683	0.07622	(1) p (ST)
HSP90AA1	3	ESEDKPEIEDVGSDEEEEKK	0.7535	0.0578	(13) p (ST)
HSP90AB1	3	IEDVGSDEEDDSGKDK	2.306	0.08044	(6) p (ST)
HSP90AB1	3	IEDVGSDEEDDSGKDKK	1.627	0.05836	(6) p (ST)
HSPD1	2	TVIIEQSWGSPK	0.9687	0.03856	(10) p (ST)
HSPD1	3	ALMLQGVDLLADAVAVTMGPK	0.7533	0.06721	(3/18) o (M)
KHDRBS1	5	ASPATQPPPLLPPSATGPDATVGGPAPTPLLPPSATASVK	0.9461	0.04577	(2) p (ST)
KIAA1429	2	SFLSEPSSPGR	1.122	0.1307	(8) p (ST)
KIF1A	3	SDSLILDHQWELEK	0.9571	0.05673	(3) p (ST)
KRI1	3	AFVEDSEDEDGAGEGGSSLLQK	0.8568	0.06957	(6) p (ST)
MAP1B	2	SDISPLTPR	0.5232	0.1389	(7) p (ST)
MAP1B	3	EVPSKEEPSPVKAEVAEK	1.175	0.04861	(9) p (ST)
MAP1B	4	KLGDVSPTQIDVSQFGSFKEDTK	0.6615	0.05714	(6) p (ST)
MAP1B	4	VLSPLRSPPLIGSESAYESFLSADDKASGR	0.7465	0.01736	(7) p (ST)
MAP1B	4	VLSPLRSPPLIGSESAYESFLSADDKASGR	0.6473	0.04706	(3/7) p (ST)
MAP1B	4	TELSPSFINPNPLEWFASEEPTEESEKPLTQSGGAPPPPGGK	0.7965	0.01479	(4) p (ST)
MAP1B	4	TELSPSFINPNPLEWFASEEPTEESEKPLTQSGGAPPPPGGK	0.7963	0.01477	(6) p (ST)
MAP1B	5	TELSPSFINPNPLEWFASEEPTEESEKPLTQSGGAPPPPGGK	0.7743	0.02306	(6) p (ST)
MAP4	3	VGSLDNVGHLPAGGAVK	0.8019	0.09142	(3) p (ST)
MATR3	4	RDSFDDRGPSLNPVLDYDHGSR	0.7213	0.01179	(3) p (ST)
MYEF2	3	AEVPGATGGDSPHLQPAEPPGEPR	1.201	0.02592	(11) p (ST)
MYH9	3	KGAGDGSDEEVDGKADGAEAKPAE	0.7672	0.05487	(7) p (ST)
NACA	4	VQGEAVSNIQENTQTPTVQEESEEEEVDETGVEVK	0.9373	0.02581	(22) p (ST)
NDRG1	2	TASGSSVTSLDGTR	0.7639	0.02355	(3) p (ST)
NIPBL	2	AITSLLGGGSPK	0.7237	0.07841	(10) p (ST)
NOL9	4	LAAFADALEFADEEKESPVEFTGHK	0.7263	0.09405	(17) p (ST)
NOLC1	3	AESSNSSSSDDSSEEEEEKLK	1.061	0.05673	(7) p (ST)
NOLC1	3	AESSNSSSSDDSSEEEEEKLK	1.061	0.05673	(6) p (ST)
NOLC1	4	SSDSDSDSSSEDEPPKNQKPK	0.9715	0.01445	(4) p (ST)
NOLC1	4	SSDSDSDSSSEDEPPKNQKPK	0.9735	0.01417	(9) p (ST)
NOLC1	3	AASSSSSSSSSSSSDDSEEEKAAATPK	0.7654	0.04805	(7) p (ST)
NOLC1	4	KASSSDSEDSSEEEEEVQGPPAKK	0.7794	0.05818	(3) p (ST)
NOLC1	3	AASSSSSSSSSSSSDDSEEEKAAATPK	0.7654	0.04805	(3) p (ST)
NOLC1	4	KASSSDSEDSSEEEEEVQGPPAKK	0.7794	0.05818	(5) p (ST)
NOLC1	4	ASESSSSEESSDDDDEEDQKKQPVQK	0.9112	0.01664	(6) p (ST)
NOLC1	4	ASESSSSEESSDDDDEEDQKKQPVQK	0.9112	0.01664	(10) p (ST)
NOLC1	4	QQPVESSEDSSDESDSSSEEEKKPPTK	0.8908	0.02328	(16) p (ST)
NOLC1	4	QQPVESSEDSSDESDSSSEEEKKPPTK	0.8908	0.02328	(17) p (ST)
NOP58	4	HIKEEPLSEEEPCTSTAIASPEK	1.331	0.1898	(8) p (ST)
NOP58	4	HIKEEPLSEEEPCTSTAIASPEKK	0.7663	0.09106	(8) p (ST)
NPM1	4	CGSGPVHISGQHLVAVEEDAESEDEEEEDVK	1.101	0.02527	(22) p (ST)
NUMA1	3	KNSLISSLEEEVSILNR	0.8451	0.07158	(3) p (ST)
NUP35	2	TLGTPTQPGSTPR	1.081	0.05633	(4) p (ST)
P4HB	4	FLESGGQDGAGDDDDLEDLEEAEEPDMEEDDDQK	3.394	0.04367	(27) o (M)
PGK1	3	ALESPERPFLAILGGAK	0.8497	0.01295	(4) p (ST)
PGRMC1	3	GDQPAASGDSDDDEPPPLPR	0.7589	0.05616	(10) p (ST)
PGRMC1	3	EGEEPTVYSDEEEPKDESAR	1.266	0.0312	(9) p (ST)
PGRMC1	4	IVRGDQPAASGDSDDDEPPPLPR	0.7134	0.02287	(10) p (ST)
PGRMC1	4	EGEEPTVYSDEEEPKDESARK	0.8134	0.04398	(9) p (ST)
PNISR	4	QRSPIALPVKQEPPQIDAVK	0.8566	0.03866	(3) p (ST)
PRKACA	3	TWTLCGTPEYLAPEIILSK	0.9055	0.02777	(3) p (ST)
PRKACA	3	GRTWTLCGTPEYLAPEIILSK	0.7148	0.02782	(5) p (ST)
PRKAR1A	3	TDSREDEISPPPPNPVVK	0.9503	0.01107	(9) p (ST)
PRPF4B	3	LCDFGSASHVADNDITPYLVSR	1.158	0.03175	(18) p (Y)
PSIP1	2	QSNASSDVEVEEK	1.026	0.1402	(6) p (ST)
RBM15	2	SLSPGGAALGYR	0.9154	0.02346	(3) p (ST)
RBM39	2	YRSPYSGPK	0.9017	0.06593	(3) p (ST)
RBM39	3	YRSPYSGPK	0.000027	0.153	(3) p (ST)
RBMX2	2	EVQAEQPSSSSPR	0.9258	0.04553	(10) p (ST)
RBMX2	2	EVQAEQPSSSSPR	0.9258	0.04553	(11) p (ST)
RCC1	3	RRSPPADAIPK	0.8344	0.01407	(3) p (ST)
RPL12	2	IGPLGLSPK	0.8161	0.034	(7) p (ST)
RPLP0	5	AFLADPSAFVAAAPVAAATTAAPAAAAAPAKVEAKEESEESDEDMGFGLFD	0.6218	0.08874	(41) p (ST)
RPLP2	3	YVASYLLAALGGNSSPSAK	1.002	0.02125	(15) p (ST)
RPLP2	2	KEESEESDDDMGFGLFD	0.765	0.02764	(11) o (M)
RPRD1B	4	TFQQIQEEEDDDYPGSYSPQDPSAGPLLTEELIK	0.7185	0.04067	(18) p (ST)
RPRD1B	5	TFQQIQEEEDDDYPGSYSPQDPSAGPLLTEELIK	0.8116	0.02616	(18) p (ST)
RPS3	2	DEILPTTPISEQK	1.258	0.03964	(7) p (ST)
SCAF4	5	IEIIQPLLDMAAGTSNAAPVAENVTNNEGSPPPPVK	0.8588	0.04668	(30) p (ST)
SLC35B2	2	AVPVESPVQKV	1.054	0.07057	(6) p (ST)
SLC35C2	2	GLGSSPDLELLLR	0.8965	0.02242	(4) p (ST)
SMARCA5	4	GGPEGVAAQAVASAASAGPADAEMEEIFDDASPGKQK	0.7985	0.1748	(32) p (ST)
SNIP1	3	QERLSPEVAPPAHR	0.8666	0.01985	(5) p (ST)
SPAG1	3	KQSLGELIGTLNAAK	0.8809	0.03595	(3) p (ST)
SPAG1	4	KQSLGELIGTLNAAKVPADTEVVCAPPTAYIDFAR	0.5266	0.05803	(10) p (ST)
SRRM1	3	HRPSPPATPPPK	0.7073	0.06718	(4) p (ST)
SRRM1	3	HRPSPPATPPPK	0.7098	0.1964	(4/8) p (ST)
SRRM1	3	EKTPELPEPSVK	0.8477	0.01698	(3) p (ST)
SRRM2	3	SSTPPGESYFGVSSLQLK	0.9402	0.04744	(3) p (ST)
SRRM2	4	RGEGDAPFSEPGTTSTQRPSSPETATK	0.9994	0.03512	(20) p (ST)
SRRM2	4	RGEGDAPFSEPGTTSTQRPSSPETATK	0.9994	0.03512	(21) p (ST)
SRRT	4	TQLWASEPGTPPLPTSLPSQNPILK	0.9309	0.05332	(10) p (ST)
SRRT	3	TQLWASEPGTPPLPTSLPSQNPILK	1.002	0.02693	(10) p (ST)
SRSF9	3	GSPHYFSPFRPY	0.9695	0.01236	(2) p (ST)
STT3B	3	ENPPVEDSSDEDDKR	1.109	0.05264	(8) p (ST)
STT3B	3	ENPPVEDSSDEDDKR	1.109	0.05264	(9) p (ST)
SUB1	3	ELVSSSSSGSDSDSEVDKK	0.8291	0.05701	(12) p (ST)
SUB1	3	ELVSSSSSGSDSDSEVDKK	0.8291	0.05701	(10) p (ST)
SUB1	3	SKELVSSSSSGSDSDSEVDKK	0.5331	0.09591	(14) p (ST)
TCEA1	3	KKEPAITSQNSPEAR	0.7926	0.02504	(11) p (ST)
TCOF1	3	TSQVGAASAPAKESPR	0.7965	0.0557	(14) p (ST)
TCOF1	3	LGAGEGGEASVSPEKTSTTSK	0.8489	0.1059	(12) p (ST)
TERF2IP	2	YLLGDAPVSPSSQK	0.3456	0.07801	(9) p (ST)
THRAP3	2	ASAVSELSPR	0.9358	0.01925	(8) p (ST)
THRAP3	2	IDISPSTFR	0.9216	0.01826	(4) p (ST)
THRAP3	2	RIDISPSTFR	1.046	0.01121	(5) p (ST)
THRAP3	3	IDISPSTFRK	0.9703	0.0301	(4) p (ST)
THRAP3	3	ERSPALKSPLQSVVVR	0.8625	0.02384	(3) p (ST)
TMPO	2	SSTPLPTISSSAENTR	1.548	0.03367	(3) p (ST)
TMPO	5	NRPPLPAGTNSKGPPDFSSDEEREPTPVLGSGAAAAGR	0.6283	0.0464	(19) p (ST)
TMX1	3	KVEEEQEADEEDVSEEEAESK	1.056	0.04561	(14) p (ST)
TP53BP1	2	STPFIVPSSPTEQEGR	0.7914	0.03368	(9) p (ST)
UBE2J1	3	RLSTSPDVIQGHQPR	0.9708	0.08069	(3) p (ST)
UBE2J1	3	RLSTSPDVIQGHQPR	0.9857	0.07487	(5) p (ST)
WRAP53	3	VFPEPTESGDEGEELGLPLLSTR	0.9362	0.01495	(8) p (ST)
ZRANB2	3	EVEDKESEGEEEDEDEDLSK	1.887	0.05523	(7) p (ST)

Table S4

IDs	Name	Diff
A2ML1	Alpha-2-macroglobulin-like 1	3
ACTB	Actin, beta	2
ADA	Adenosine deaminase	1
AKAP8	A kinase (PRKA) anchor protein 8	3
AKR1B10	Aldo-keto reductase family 1, member B10	-3
ALB	Albumin	15
ALDOA	Aldolase A, fructose-bisphosphate	-2
ANGEL2	Angel homolog 2	0
ANXA1	Annexin A1	-3
ANXA2	Annexin A2	1
ARF3	ADP-ribosylation factor 3	0
ARG1	Arginase, liver	-4
ASPRV1	Aspartic peptidase, retroviral-like 1	1
ATP5A1	ATP ∑ase, H+ transporting, mito F1 complex, alpha 1	3
ATP5B	ATP ∑ase, mitochon F1 complex, beta polypep	1
B2R5S5	Ser-Thr kinase receptor-associated protein	-8
B2RNR6	Zinc finger RNA binding protein	1
C14orf166	Chromosome 14 open reading frame 166	-3
C1orf68	Chromosome 1 open reading frame 68	2
C22orf28	Chromosome 22 open reading frame 28	-2
C3	Complement component 3	4
CALML3	Calmodulin-like 3	-2
CALML5	Calmodulin-like 5	-1
CANX	Calnexin	0
CAPRIN1	Cell cycle associated protein 1	5
CDKN2AIF	CDKN2A interacting protein	-1
CFL1	Cofilin 1	-3
CKMT1A	Creatine kinase, mitochondrial 1A	1
CLTC	Clathrin, heavy chain (Hc)	0
CSDA	Cold shock domain protein A	-1
CTSD	Cathepsin D	6
DAP3	Death associated protein 3	0
DCD	Dermcidin	1
DDX1	DEAD box polypeptide 1	1
DDX17	DEAD box polypeptide 17	2
DDX28	DEAD box polypeptide 28	0
DDX3X	DEAH box polypeptide 3, X-linked	0
DDX5	DEAH box polypeptide 5	0
DDX6	DEAD box polypeptide 6	-3
DHX15	DEAH box polypeptide 15	6
DHX30	DEAH box polypeptide 30	-1
DHX36	DEAH box polypeptide 36	2
DHX9	DEAH box polypeptide 9	1
DIMT1L	DIM1 dimethyladenosine transferase 1-like	2
DNAJC10	DnaJ (Hsp40) homolog, subfamily C, member 10	2
DSG1	Desmoglein 1	3
DSP	Desmoplakin	2
EEF1A1	Eukaryotic translation elongation factor 1 alpha 1	5
EEF1G	Eukaryotic translation elongation factor 1 gamma	0
EEF2	Eukaryotic translation elongation factor 2	1
EIF2B1	Eukaryotic translation initiation factor 2B, subu 1 alpha	-5
EIF2B3	eukaryotic translation initiation factor 2B, subu 3 gamma	1
EIF3H	Eukaryotic translation initiation factor 3, subu H	0
EIF4A2	Eukaryotic translation initiation factor 4A2	0
EIF4A3	Eukaryotic translation initiation factor 4A3	1
ELAVL1	ELAV-like 1 (Hu antigen R)	2
ENO1	Enolase 1	-5
EPPK1	Epiplakin 1	-6
EWSR1	Ewing sarcoma breakpoint region 1	0
FAM120A	Family with sequence similarity 120A	7
FAM120C	Family with sequence similarity 120C	-1
FAM35A	Family with sequence similarity 35, member A	-1
FAM98B	Family with sequence similarity 98, member B	2
FLG	Filaggrin	-5
FLG2	Filaggrin family member 2	2
FLNA	Filamin A, alpha	1
FUS	Fused in sarcoma	-3
G3BP1	GTPase activating protein binding protein 1	-1
G3BP2	GTPase activating protein binding prot 2	1
GADD45G	Growth arrest/DNA-damage-inducible, γ interac prot 1	1
GAPDH	Glyceraldehyde-3-phosphate dehydrogenase	0
GDE1	Glycerophosphodiester phosphodiesterase 1	-1
GM2A	GM2 ganglioside activator	0
GNB2L1	G prot, beta polypeptide 2-like 1	5
GSN	Gelsolin	3
GSTP1	Glutathione S-transferase pi 1	-5
GTF2I	General transcription factor II-I	4
H1FX	H1 histone family, member X	-1
H3F3A	H3 histone, family 3A	0
HAL	Histidine ammonia-lyase	-2
HIST1H1E	Histone cluster 1, H1e	-2
HIST1H2A	Histone cluster 1, H2ab	-1
HNRNPA0	Heterogeneous nuclear ribonucleoprotein A0	0
HNRNPA2	Heterogeneous nuclear ribonucleoprotein A2/B1	2
HNRNPA3	Heterogeneous nuclear ribonucleoprotein A3	-1
HNRNPAB	Heterogeneous nuclear ribonucleoprotein A/B	-2
HNRNPC	Heterogeneous nuclear ribonucleoprotein C (C1/C2)	5
HNRNPD	Heterogeneous nuclear ribonucleoprotein D	1
HNRNPF	Heterogeneous nuclear ribonucleoprotein F	-1
HNRNPH1	Heterogeneous nuclear ribonucleoprotein H1 (H)	0
HNRNPH2	Heterogeneous nuclear ribonucleoprotein H2 (H')	0
HNRNPH3	Heterogeneous nuclear ribonucleoprotein H3	0
HNRNPK	Heterogeneous nuclear ribonucleoprotein K	1
HNRNPL	Heterogeneous nuclear ribonucleoprotein L	0
HNRNPM	Heterogeneous nuclear ribonucleoprotein M	5
HNRNPR	Heterogeneous nuclear ribonucleoprotein R	3
HNRNPU	Heterogeneous nuclear ribonucleoprotein U	-3
HNRNPUL	Heterogeneous nuclear ribonucleoprotein U-like 1	-1
HNRNPUL	Heterogeneous nuclear ribonucleoprotein U-like 2	3
HNRPDL	Heterogeneous nuclear ribonucleoprotein D-like	0
HRNR	Hornerin	5
HSP90AB1	Heat shock protein 90kDa alpha, class B member 1	-6
HSPA1A	Heat shock 70kDa protein 1A	-3
HSPA5	Heat shock 70kDa protein 5	-3
HSPA8	Heat shock 70kDa protein 8 isoform 1	-3
HSPB1	Heat shock 27kDa protein 1	-5
HSPD1	Heat shock 60kDa protein 1	0
ICT1	Immature colon carcinoma transcript 1	1
IGF2BP2	Insulin-like growth factor 2 mRNA binding protein 2	0
IGH@ prot	IGH@ protein	4
IGK@ prot	IGK@ protein	6
IGL@	IGL@	2
IL36G	Interleukin 36, gamma	-2
ILF2	interleukin enhancer binding factor 2	-2
ILF3	Interleukin enhancer binding factor 3	3
IVL	Involucrin	0
JUP	Junction plakoglobin	8
KHSRP	KH-type splicing regulatory protein	2
KIAA1967	KIAA1967	-2
KPRP	Keratinocyte proline-rich protein	-1
KRT1	Keratin 1	-1
KRT10	Keratin 10	-1
KRT14	Keratin 14	5
KRT15	Keratin 15	2
KRT16	Keratin 16	0
KRT17	Keratin 17	3
KRT2	Keratin 2	8
KRT33A	Keratin 33A	3
KRT34	Keratin 34	-2
KRT5	Keratin 5	-3
KRT6A	Keratin 6A	0
KRT6B	keratin 6B	-1
KRT6B	Keratin 6B	0
KRT6C	Keratin 6C	4
KRT6C	Keratin 6C	-2
KRT78	Keratin 78	1
KRT79	Keratin 79	7
KRT80	Keratin 80	-2
KRT83	keratin 83	13
KRT9	Keratin 9	4
KV303	Ig kappa chain V-III region NG9	0
LAMP1	Lysosomal-associated membrane protein 1	2
LARP1	La ribonucleoprotein domain family, member 1	-1
LARP3	Autoantigen La	0
LARP7	La ribonucleoprotein domain family, member 7	1
LCN2	Lipocalin 2	3
LDHA	Lactate dehydrogenase A	1
LGALS7	Lectin, galactoside-binding, soluble, 7	1
LMNA	lamin A/C	1
LRPPRC	Leucine-rich PPR-motif containing	-4
LTV1	LTV1 homolog	1
MARK2	MAP/microtubule affinity-regulating kinase 2	1
MATR3	Matrin 3	2
MOV10	Mov10, Moloney leukemia virus 10, homolog	4
MRPL1	Mitochondrial ribosomal protein L1	0
MRPL11	Mitochondrial ribosomal protein L11	1
MRPL15	Mitochondrial ribosomal protein L15	-1
MRPL18	Mitochondrial ribosomal protein L18	0
MRPL19	Mitochondrial ribosomal protein L19	-2
MRPL22	Mitochondrial ribosomal protein L22	-1
MRPL24	Mitochondrial ribosomal protein L24	0
MRPL3	Mitochondrial ribosomal protein L3	1
MRPL37	Mitochondrial ribosomal protein L37	-3
MRPL38	Mitochondrial ribosomal protein L38	0
MRPL39	Mitochondrial ribosomal protein L39	-1
MRPL43	Mitochondrial ribosomal protein L43	2
MRPL44	Mitochondrial ribosomal protein L44	-1
MRPL45	Mitochondrial ribosomal protein L45	-1
MRPL46	Mitochondrial ribosomal protein L46	-1
MRPL48	Mitochondrial ribosomal protein L48	1
MRPS15	Mitochondrial ribosomal protein S15	0
MRPS18B	Mitochondrial ribosomal protein S18B	-1
MRPS2	Mitochondrial ribosomal protein S2	0
MRPS22	Mitochondrial ribosomal protein S22	-7
MRPS23	Mitochondrial ribosomal protein S23	-1
MRPS27	mitochondrial ribosomal protein S27	-4
MRPS28	Mitochondrial ribosomal protein S28	0
MRPS30	Mitochondrial ribosomal protein S30	0
MRPS31	Mitochondrial ribosomal protein S31	-2
MRPS35	Mitochondrial ribosomal protein S35	-2
MRPS5	Mitochondrial ribosomal protein S5	-2
MRPS7	Mitochondrial ribosomal protein S7	-1
MRPS9	Mitochondrial ribosomal protein S9	-1
MX1	myxovirus resist 1, interferon-inducible protein p78	-4
MYH9	Myosin, heavy chain 9, non-muscle	5
MYL12A	Myosin, light chain 12A, regulatory, non-sarcomeric	-2
NAMPT	Pre-B-cell colony enhancing factor 1	-3
NCCRP1	Non-specific cytotoxic cell receptor prot 1 homolog	-2
NCL	Nucleolin	-1
NGRN	Neugrin, neurite outgrowth associated	2
NONO	Non-POU domain containing, octamer-binding	-4
NSUN5	NOP2/Sun domain family, member 5	2
NXF1	Nuclear RNA export factor 1	2
P4HB	Prolyl 4-hydroxylase, beta polypeptide	-7
PABPC1	Poly(A) binding protein, cytoplasmic 1	-2
PCBP2	Poly(rC) binding protein 2	-4
PFN1	Profilin 1	-1
PGK1	Phosphoglycerate kinase 1	-5
PHB2	Prohibitin 2	2
PICALM	Phosphatidylinositol binding clathrin assembly protein	-4
PKM2	Pyruvate kinase, muscle	-3
PKP1	Plakophilin 1	7
PLOD1	Procollagen-lysine 1, 2-oxoglutarate 5-dioxygenase 1	2
PLOD1	procollagen-lysine 1, 2-oxoglutarate 5-dioxygenase 1	-1
PNO1	Partner of NOB1 homolog	-2
PNP	Purine nucleoside phosphorylase	-1
POF1B	Premature ovarian failure, 1B	-1
POLDIP3	DNA polymerase, delta interacting prot 3	1
PPIA	Peptidylprolyl isomerase A	-2
PPL	Periplakin	-2
PPP2R1A	Prot phosphatase 2, regulatory subunit A, alpha	2
PRDX1	Peroxiredoxin 1	3
PRMT1	Protein arginine methyltransferase 1	-1
PRPF19	PRP19/PSO4 pre-mRNA processing factor 19 homolog	1
PRPF31	PRP31 pre-mRNA processing factor 31 homolog	0
PRSS3	Protease, serine, 3	0
PTBP1	Polypyrimidine tract binding protein 1	0
PTCD3	Pentatricopeptide repeat domain 3	-2
Q2I377	Small proline rich protein	0
Q5K634	SCCA2/SCCA1 fusion protein isoform 1	-8
RAB5A	RAB5A, member RAS oncogene family	-2
RALY	RNA binding protein, autoantigenic	1
RBM14	RNA binding motif protein 14	2
RBM45	RNA binding motif protein 45	4
RBM6	RNA binding motif protein 6	6
RBMX	Heterogeneous nuclear ribonucleoprotein G	0
RNMT	RNA (guanine-7-) methyltransferase	0
RPL10	Ribosomal protein L10	0
RPL11	Ribosomal protein L11	1
RPL13	Ribosomal protein L13	4
RPL15	Ribosomal protein L15	-1
RPL17	Ribosomal protein L17	1
RPL19	Ribosomal protein L19	1
RPL23	Ribosomal protein L23	0
RPL23A	Ribosomal protein L23a	0
RPL24	Ribosomal protein L24	-1
RPL26	Ribosomal protein L26	3
RPL27A	Ribosomal protein L27a	2
RPL3	Ribosomal protein L3	1
RPL31	Ribosomal protein L31	1
RPL4	Ribosomal protein L4	0
RPL6	Ribosomal protein L6	-3
RPL7	Ribosomal protein L7	1
RPL7A	Ribosomal protein L7a	-1
RPL8	Ribosomal protein L8	1
RPLP0	Ribosomal protein, large, P0	-1
RPS10	ribosomal protein S10	-2
RPS11	Ribosomal protein S11	3
RPS13	ribosomal protein S13	2
RPS15A	Ribosomal protein S15a	0
RPS16	Ribosomal protein S16	1
RPS17	Ribosomal protein S17	3
RPS18	Ribosomal protein S18	0
RPS19	Ribosomal protein S19	1
RPS2	Ribosomal protein S2	-1
RPS20	Ribosomal protein S20	-1
RPS25	Ribosomal protein S25	0
RPS28	Ribosomal protein S28	0
RPS3	Ribosomal protein S3	2
RPS3A	Ribosomal protein S3A	-4
RPS4X	Ribosomal protein S4, X-linked	4
RPS5	Ribosomal protein S5	0
RPS6	Ribosomal protein S6	-1
RPS6KA1	Ribosomal protein S6 kinase, 90kDa, polypeptide 1	-3
RPS7	Ribosomal protein S7	2
RPS8	Ribosomal protein S8	2
RPS9	Ribosomal protein S9	2
RPSA	Ribosomal protein SA	1
S100A11	S100 calcium binding protein A11	-2
S100A14	S100 calcium binding protein A14	-1
S100A16	S100 calcium binding protein A16	3
S100A2	S100 calcium binding protein A2	2
S100A7	S100 calcium binding protein A7	1
S100A8	S100 calcium binding protein A8	-1
S100A9	S100 calcium binding protein A9	0
SAE1	SUMO1 activating enzyme subunit 1	-3
SERBP1	SERPINE1 mRNA binding protein 1	-1
SERPINB3	Serpin peptidase inhibitor, clade B , member 3	-5
SERPINB5	Serpin peptidase inhibitor, clade B, member 5	-2
SF3B2	Splicing factor 3b, subunit 2	3
SFN	14-3-3 protein sigma	-6
SFPQ	Splicing factor proline/glutamine-rich	6
SKIV2L2	superkiller viralicidic activity 2-like 2	4
SLC25A10	39S ribosomal protein L12, mitochondrial carrier	-1
SND1	Staphylococcal nuclease/tudor domain containing 1	-2
SPRR2D	Small proline-rich protein 2D	2
SRSF1	Serine/arginine-rich splicing factor 1	2
SRSF10	Serine/arginine-rich splicing factor 10	2
SRSF3	Serine/arginine-rich splicing factor 3	-1
SRSF6	Serine/arginine-rich splicing factor 6	0
SRSF9	Serine/arginine-rich splicing factor 9	5
STAU1	Staufen, RNA binding protein, homolog 1	-2
SYNCRIP	Heterogeneous nuclear ribonucleoprotein Q	-2
TAGLN2	Transgelin 2	-1
TARDBP	TAR DNA-binding prot	-1
TF	Transferrin	2
TFAM	Transcription factor A, mitochondrial	2
TGM1	Transglutaminase 1	1
TGM3	Transglutaminase 3	-7
THOC4	THO complex 4	0
TPI1	Triosephosphate isomerase 1	-3
TRIM29	Tripartite motif containing 29	0
TRUB2	TruB pseudouridine (psi) synthase homolog 2	-2
TUBA1B	Tubulin, alpha 1b	-3
TUBA4A	Tubulin, alpha 4a	1
TUBB	Tubulin, beta	2
TUBB2C	Tubulin, beta 2C	1
TUBB4	Tubulin, beta 4	1
TYMP	Thymidine phosphorylase	-1
UBA2	Ubiquitin-like modifier activating enzyme 2	-1
UPF1	UPF1 regulator of nonsense transcripts homolog	-1
VCP	Valosin containing protein	1
WBSCR22	Williams Beuren syndrome chromosome region 22	-1
WDR6	WD repeat domain 6	2
WIBG	Within bgcn homolog	1
XRCC5	X-ray repair complem defective in Chinese hamster cells 5	3
XRCC6	X-ray repair complem defective in Chinese hamster cells 6	1
XRN2	5'-3' exoribonuclease 2	0
YBX1	Y box binding protein 1	-3
YTHDC2	YTH domain containing 2	-2
YWHAE	14-3-3 epsilon	-1
YWHAQ	Tyr 3-/Trp 5-monooxygenase activation prot, theta	1
YWHAZ	Tyr 3-/Trp 5-monooxygenase activation prot, zeta polypep	1
ZC3HAV1	Zinc finger CCCH-type, antiviral 1	2
ZNF326	Zinc finger protein 326	-2

Figure S1

A

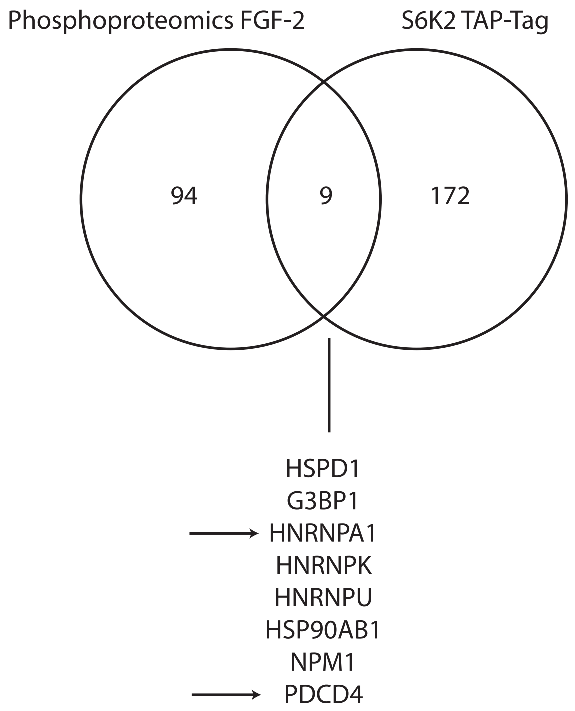

B

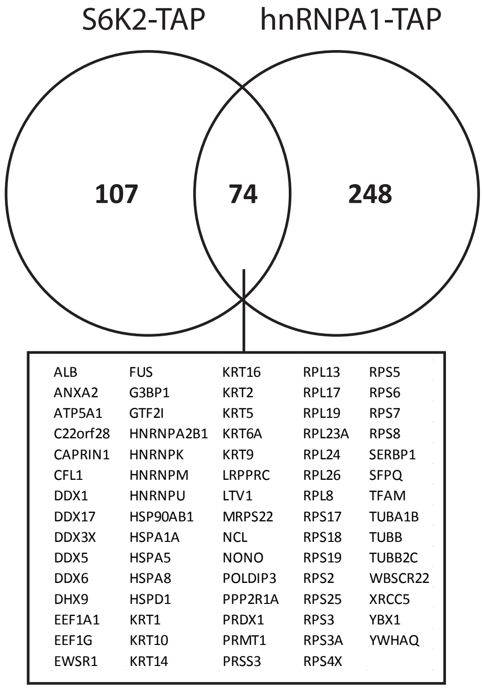

C

GO annotation	Q-value	Genes in network	Genes in genome
**Translation-related**			
Translational elongation	2.30E-69	41	97
Ribosome	9.23E-59	40	150
Cytosolic ribosome	4.11E-67	39	88
Translational termination	4.11E-67	39	88
Structural constituent of ribosome	3.78E-43	29	97
Small ribosomal subunit	4.37E-33	21	55
Cytosolic small ribosomal subunit	5.26E-36	20	35
Cytosolic large ribosomal subunit	1.85E-29	19	51
Large ribosomal subunit	4.29E-27	19	65
Ribonucleoprotein complex biogenesis	3.11E-11	13	135
Cellular component biogenesis at cellular level	5.82E-11	13	142
Ribosome biogenesis	7.22E-14	12	63
Ribosomal small subunit biogenesis	4.15E-10	7	17
Regulation of translation	3.69E-03	6	106
Translational initiation	4.99E-03	5	66
Ribosomal large subunit biogenesis	6.36E-05	4	11
Cytoplasmic stress granule	5.37E-04	4	18
ATP-dependent RNA helicase activity	2.99E-02	3	20
**RNA processing**			
ncRNA processing	2.20E-11	12	100
ncRNA metabolic process	1.86E-08	12	177
RNA splicing	9.13E-07	12	250
rRNA processing	3.20E-13	11	51
rRNA metabolic process	9.45E-13	11	56
Posttranscriptional regulation of gene expression	1.79E-06	11	210
RNA splicing, via transesterification reactions with bulged adenosine as nucleophile	1.13E-05	10	196
Nuclear mRNA splicing, via spliceosome	1.13E-05	10	196
RNA splicing, via transesterification reactions	1.46E-05	10	202
mRNA processing	1.22E-04	10	256
RNA helicase activity	2.27E-08	7	28
Spliceosomal complex	4.25E-03	6	109
mRNA binding	5.98E-03	5	69
Catalytic step 2 spliceosome	1.19E-02	5	80
Nuclear body	7.21E-02	5	123
RNA-dependent ATPase activity	1.02E-03	4	21
Regulation of mRNA stability	1.71E-03	4	24
Regulation of RNA stability	1.99E-03	4	25
Ribonucleoprotein granule	2.28E-02	4	49
Alternative nuclear mRNA splicing, via spliceosome	1.88E-02	3	17
RNA stabilization	1.88E-02	3	17
Heterogeneous nuclear ribonucleoprotein complex	1.88E-02	3	17
mRNA stabilization	1.88E-02	3	17
**Protein complex**			
Cellular protein complex disassembly	3.91E-61	39	118
Protein complex disassembly	1.32E-60	39	122
Cellular macromolecular complex disassembly	2.63E-58	39	138
Macromolecular complex disassembly	8.69E-58	39	142
Cellular component disassembly at cellular level	3.28E-50	39	214
Cellular component disassembly	5.59E-50	39	217
Signalosome	8.78E-02	3	29
**Others**			
Cytosolic part	7.35E-61	40	134
Viral infectious cycle	5.37E-52	40	215
Viral reproductive process	2.86E-50	40	236
Endocrine pancreas development	3.28E-61	39	117
Pancreas development	1.69E-60	39	123
Endocrine system development	1.75E-57	39	145
Viral transcription	1.75E-57	39	145
Viral genome expression	1.75E-57	39	145
ATPase activity	1.06E-04	9	191
Intermediate filament	3.99E-07	7	41
Structural constituent of cytoskeleton	9.03E-06	7	64
Intermediate filament cytoskeleton	1.88E-05	7	72
Epidermis development	1.34E-03	7	137
Helicase activity	7.40E-06	7	62
ATPase activity, coupled	1.82E-02	6	145
Protein folding	4.59E-02	5	111
Response to unfolded protein	1.30E-02	4	41
Purine NTP-dependent helicase activity	1.38E-02	4	42
ATP-dependent helicase activity	1.38E-02	4	42
Response to topologically incorrect protein	1.88E-02	4	46
Cytoskeleton-dependent intracellular transport	9.59E-02	3	30
MHC class I protein binding	5.71E-03	3	11
Telomeric DNA binding	1.19E-02	3	14
MHC protein binding	2.58E-02	3	19
Chaperone binding	7.21E-02	3	27

Figure S2

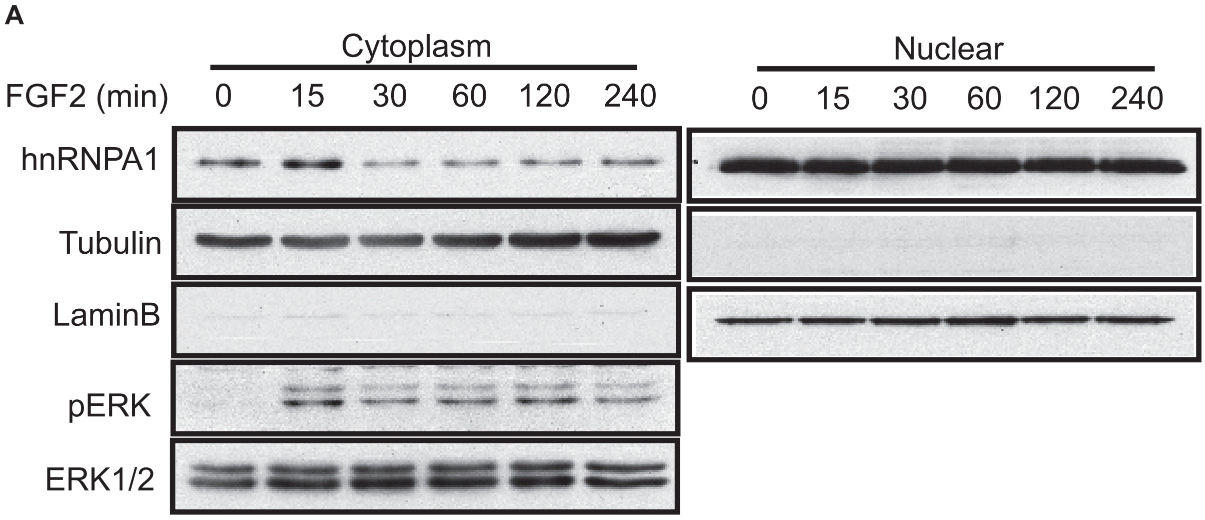

A
Cytoplasm
Nuclear
FGF2 (min)
0
15
30
60
120
240
hnRNPA1
Tubulin
LaminB
pERK
ERK1/2

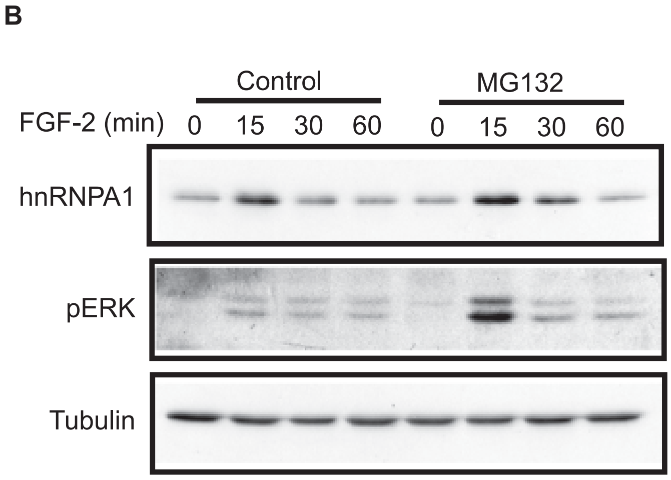

B
Control
MG132
FGF-2 (min)
0
15
30
60
hnRNPA1
pERK
Tubulin

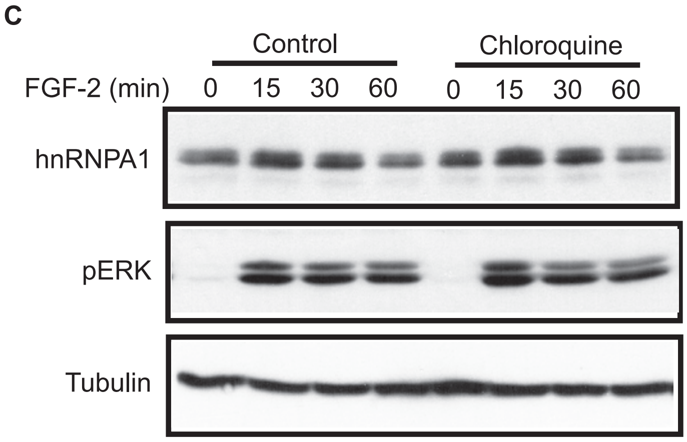

C
Control
Chloroquine
FGF-2 (min)
0
15
30
60
hnRNPA1
pERK
Tubulin

Figure S3

A

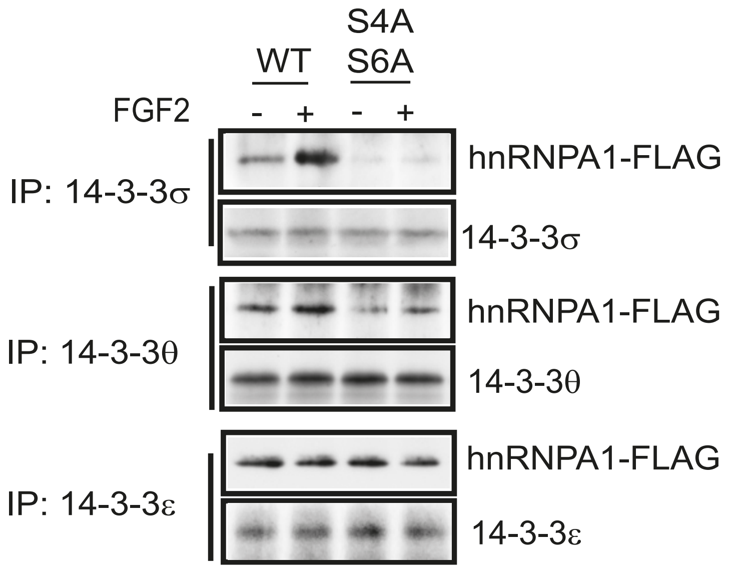

B

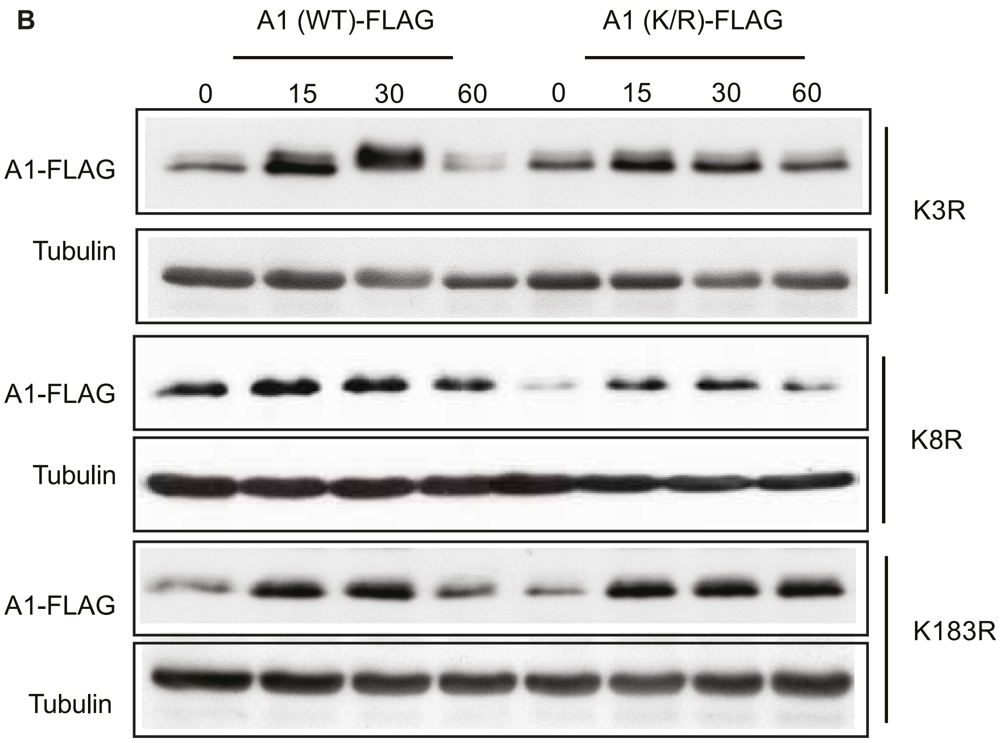

C

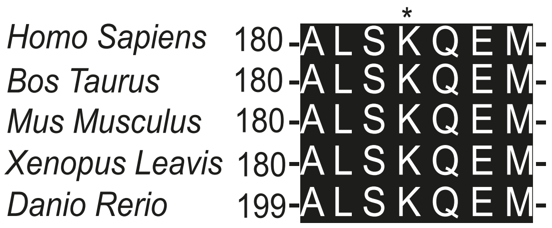

Figure S4

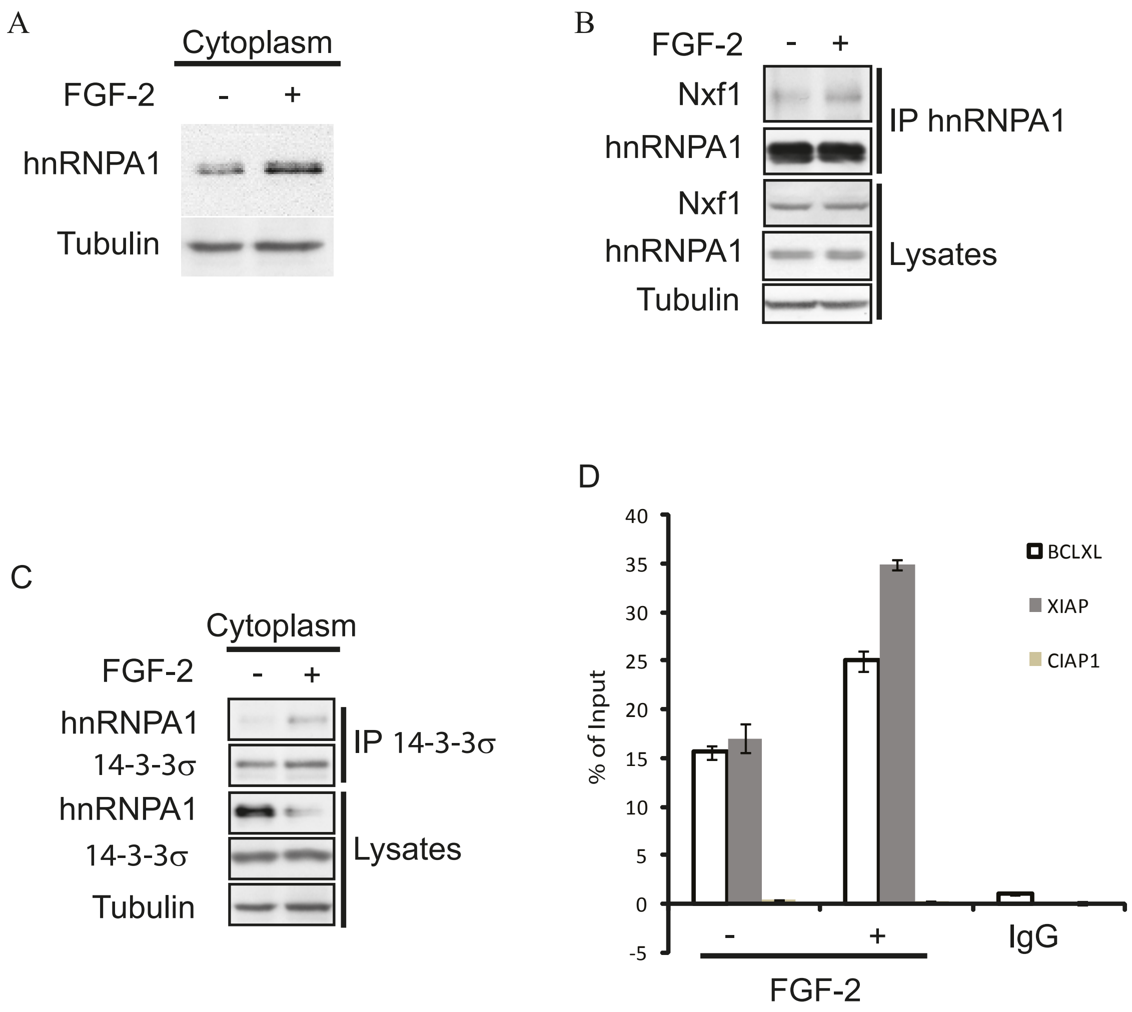
A
Cytoplasm
FGF-2
-
+
hnRNPA1
Tubulin
B
FGF-2
-
+
Nxf1
hnRNPA1
IP hnRNPA1
Nxf1
hnRNPA1
Tubulin
Lysates
C
Cytoplasm
FGF-2
-
+
hnRNPA1
14-3-3σ
IP 14-3-3σ
hnRNPA1
14-3-3σ
Tubulin
Lysates
D
% of Input
40
35
30
25
20
15
10
5
0
-5
BCLXL
XIAP
CIAP1
-
+
IgG
FGF-2

Figure S5

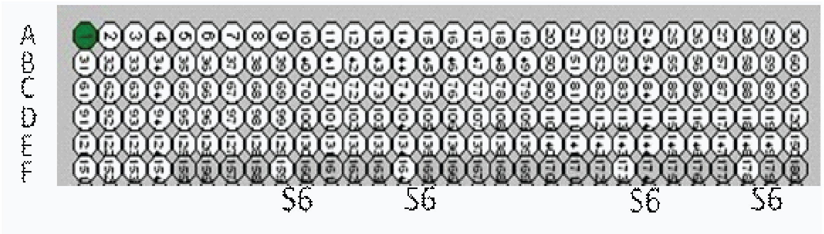

Nr.	Pos.	Mol.Weight	Sequence Label
1	A 1	1645.9	M-S-K-S-E-S-P-K-E-P-E-Q-L-R
2	A 2	1669	K-S-E-S-P-K-E-P-E-Q-L-R-K-L
3	A 3	1714.1	E-S-P-K-E-P-E-Q-L-R-K-L-F-I
4	A 4	1612.1	P-K-E-P-E-Q-L-R-K-L-F-I-G-G
5	A 5	1587.1	E-P-E-Q-L-R-K-L-F-I-G-G-L-S
6	A 6	1637.2	E-Q-L-R-K-L-F-I-G-G-L-S-F-E
7	A 7	1582.2	L-R-K-L-F-I-G-G-L-S-F-E-T-T
8	A 8	1557	K-L-F-I-G-G-L-S-F-E-T-T-D-E
9	A 9	1515.9	F-I-G-G-L-S-F-E-T-T-D-E-S-L
10	A10	1498.8	G-G-L-S-F-E-T-T-D-E-S-L-R-S
11	A11	1668.9	L-S-F-E-T-T-D-E-S-L-R-S-H-F
12	A12	1725.8	F-E-T-T-D-E-S-L-R-S-H-F-E-Q
13	A13	1692.8	T-T-D-E-S-L-R-S-H-F-E-Q-W-G
14	A14	1704.9	D-E-S-L-R-S-H-F-E-Q-W-G-T-L
15	A15	1676.9	S-L-R-S-H-F-E-Q-W-G-T-L-T-D
16	A16	1678.8	R-S-H-F-E-Q-W-G-T-L-T-D-C-V
17	A17	1665.8	H-F-E-Q-W-G-T-L-T-D-C-V-V-M
18	A18	1652.8	E-Q-W-G-T-L-T-D-C-V-V-M-R-D
19	A19	1606.8	W-G-T-L-T-D-C-V-V-M-R-D-P-N
20	A20	1592.8	T-L-T-D-C-V-V-M-R-D-P-N-T-K
21	A21	1621.8	T-D-C-V-V-M-R-D-P-N-T-K-R-S
22	A22	1618.9	C-V-V-M-R-D-P-N-T-K-R-S-R-G
23	A23	1621	V-M-R-D-P-N-T-K-R-S-R-G-F-G
24	A24	1637	R-D-P-N-T-K-R-S-R-G-F-G-F-V
25	A25	1630	P-N-T-K-R-S-R-G-F-G-F-V-T-Y
26	A26	1591	T-K-R-S-R-G-F-G-F-V-T-Y-A-T
27	A27	1589.9	R-S-R-G-F-G-F-V-T-Y-A-T-V-E
28	A28	1574.8	R-G-F-G-F-V-T-Y-A-T-V-E-E-V
29	A29	1547.7	F-G-F-V-T-Y-A-T-V-E-E-V-D-A
30	A30	1545.7	F-V-T-Y-A-T-V-E-E-V-D-A-A-M
31	B 1	1484.6	T-Y-A-T-V-E-E-V-D-A-A-M-N-A
32	B 2	1473.6	A-T-V-E-E-V-D-A-A-M-N-A-R-P
33	B 3	1566.7	V-E-E-V-D-A-A-M-N-A-R-P-H-K
34	B 4	1552.7	E-V-D-A-A-M-N-A-R-P-H-K-V-D
35	B 5	1537.8	D-A-A-M-N-A-R-P-H-K-V-D-G-R
36	B 6	1549.8	A-M-N-A-R-P-H-K-V-D-G-R-V-V
37	B 7	1573.7	N-A-R-P-H-K-V-D-G-R-V-V-E-P
38	B 8	1672.9	R-P-H-K-V-D-G-R-V-V-E-P-K-R
39	B 9	1589.8	H-K-V-D-G-R-V-V-E-P-K-R-A-V
40	B10	1567.8	V-D-G-R-V-V-E-P-K-R-A-V-S-R
41	B11	1597.8	G-R-V-V-E-P-K-R-A-V-S-R-E-D
42	B12	1599.7	V-V-E-P-K-R-A-V-S-R-E-D-S-Q
43	B13	1654.8	E-P-K-R-A-V-S-R-E-D-S-Q-R-P
44	B14	1556.8	K-R-A-V-S-R-E-D-S-Q-R-P-G-A
45	B15	1522.7	A-V-S-R-E-D-S-Q-R-P-G-A-H-L
46	B16	1552.7	S-R-E-D-S-Q-R-P-G-A-H-L-T-V
47	B17	1565.8	E-D-S-Q-R-P-G-A-H-L-T-V-K-K
48	B18	1582	S-Q-R-P-G-A-H-L-T-V-K-K-I-F
49	B19	1523	R-P-G-A-H-L-T-V-K-K-I-F-V-G
50	B20	1440	G-A-H-L-T-V-K-K-I-F-V-G-G-I
51	B21	1569.1	H-L-T-V-K-K-I-F-V-G-G-I-K-E
52	B22	1535	T-V-K-K-I-F-V-G-G-I-K-E-D-T
53	B23	1593	K-K-I-F-V-G-G-I-K-E-D-T-E-E
54	B24	1610.8	I-F-V-G-G-I-K-E-D-T-E-E-H-H
55	B25	1619.8	V-G-G-I-K-E-D-T-E-E-H-H-L-R
56	B26	1741.9	G-I-K-E-D-T-E-E-H-H-L-R-D-Y
57	B27	1847.9	K-E-D-T-E-E-H-H-L-R-D-Y-F-E
58	B28	1881.9	D-T-E-E-H-H-L-R-D-Y-F-E-Q-Y
59	B29	1851	E-E-H-H-L-R-D-Y-F-E-Q-Y-G-K
60	B30	1835.1	H-H-L-R-D-Y-F-E-Q-Y-G-K-I-E
61	C 1	1773.2	L-R-D-Y-F-E-Q-Y-G-K-I-E-V-I
62	C 2	1746.1	D-Y-F-E-Q-Y-G-K-I-E-V-I-E-I
63	C 3	1700.1	F-E-Q-Y-G-K-I-E-V-I-E-I-M-T
64	C 4	1695.1	Q-Y-G-K-I-E-V-I-E-I-M-T-D-R
65	C 5	1548	G-K-I-E-V-I-E-I-M-T-D-R-G-S
66	C 6	1548	I-E-V-I-E-I-M-T-D-R-G-S-G-K
67	C 7	1590.1	V-I-E-I-M-T-D-R-G-S-G-K-K-R
68	C 8	1582.1	E-I-M-T-D-R-G-S-G-K-K-R-G-F
69	C 9	1558.1	M-T-D-R-G-S-G-K-K-R-G-F-A-F
70	C10	1526	D-R-G-S-G-K-K-R-G-F-A-F-V-T
71	C11	1517	G-S-G-K-K-R-G-F-A-F-V-T-F-D
72	C12	1625	G-K-K-R-G-F-A-F-V-T-F-D-D-H
73	C13	1641.9	K-R-G-F-A-F-V-T-F-D-D-H-D-S
74	C14	1571.7	G-F-A-F-V-T-F-D-D-H-D-S-V-D
75	C15	1608.8	A-F-V-T-F-D-D-H-D-S-V-D-K-I
76	C16	1602.8	V-T-F-D-D-H-D-S-V-D-K-I-V-I
77	C17	1658.9	F-D-D-H-D-S-V-D-K-I-V-I-Q-K
78	C18	1696.9	D-H-D-S-V-D-K-I-V-I-Q-K-Y-H
79	C19	1644.9	D-S-V-D-K-I-V-I-Q-K-Y-H-T-V
80	C20	1613.9	V-D-K-I-V-I-Q-K-Y-H-T-V-N-G
81	C21	1650.9	K-I-V-I-Q-K-Y-H-T-V-N-G-H-N
82	C22	1641.7	V-I-Q-K-Y-H-T-V-N-G-H-N-C-E
83	C23	1684.7	Q-K-Y-H-T-V-N-G-H-N-C-E-V-R
84	C24	1627.7	Y-H-T-V-N-G-H-N-C-E-V-R-K-A
85	C25	1527.7	T-V-N-G-H-N-C-E-V-R-K-A-L-S
86	C26	1583.8	N-G-H-N-C-E-V-R-K-A-L-S-K-Q
87	C27	1672.9	H-N-C-E-V-R-K-A-L-S-K-Q-E-M
88	C28	1579.9	C-E-V-R-K-A-L-S-K-Q-E-M-A-S
89	C29	1505.9	V-R-K-A-L-S-K-Q-E-M-A-S-A-S
90	C30	1424.8	K-A-L-S-K-Q-E-M-A-S-A-S-S-S
91	D 1	1509.8	L-S-K-Q-E-M-A-S-A-S-S-S-Q-R
92	D 2	1522.8	K-Q-E-M-A-S-A-S-S-S-Q-R-G-R
93	D 3	1410.7	E-M-A-S-A-S-S-S-Q-R-G-R-S-G
94	D 4	1294.6	A-S-A-S-S-S-Q-R-G-R-S-G-S-G
95	D 5	1397.7	A-S-S-S-Q-R-G-R-S-G-S-G-N-F
96	D 6	1353.7	S-S-Q-R-G-R-S-G-S-G-N-F-G-G
97	D 7	1392.8	Q-R-G-R-S-G-S-G-N-F-G-G-G-R
98	D 8	1222.7	G-R-S-G-S-G-N-F-G-G-G-R-G-G
99	D 9	1213.7	S-G-S-G-N-F-G-G-G-R-G-G-G-F
100	D10	1183.7	S-G-N-F-G-G-G-R-G-G-G-F-G-G
101	D11	1268.7	N-F-G-G-G-R-G-G-G-F-G-G-N-D
102	D12	1268.7	G-G-G-R-G-G-G-F-G-G-N-D-N-F
103	D13	1367.8	G-R-G-G-G-F-G-G-N-D-N-F-G-R
104	D14	1268.7	G-G-G-F-G-G-N-D-N-F-G-R-G-G
105	D15	1415.8	G-F-G-G-N-D-N-F-G-R-G-G-N-F
106	D16	1355.7	G-G-N-D-N-F-G-R-G-G-N-F-S-G
107	D17	1454.8	N-D-N-F-G-R-G-G-N-F-S-G-R-G
108	D18	1429.9	N-F-G-R-G-G-N-F-S-G-R-G-G-F
109	D19	1282.8	G-R-G-G-N-F-S-G-R-G-G-F-G-G
110	D20	1312.8	G-G-N-F-S-G-R-G-G-F-G-G-S-R
111	D21	1312.8	N-F-S-G-R-G-G-F-G-G-S-R-G-G
112	D22	1165.7	S-G-R-G-G-F-G-G-S-R-G-G-G-G
113	D23	1241.8	R-G-G-F-G-G-S-R-G-G-G-G-Y-G
114	D24	1172.7	G-F-G-G-S-R-G-G-G-G-Y-G-G-S
115	D25	1140.6	G-G-S-R-G-G-G-G-Y-G-G-S-G-D
116	D26	1246.7	S-R-G-G-G-G-Y-G-G-S-G-D-G-Y
117	D27	1174.6	G-G-G-G-Y-G-G-S-G-D-G-Y-N-G
118	D28	1264.7	G-G-Y-G-G-S-G-D-G-Y-N-G-F-G
119	D29	1379.7	Y-G-G-S-G-D-G-Y-N-G-F-G-N-D
120	D30	1303.6	G-S-G-D-G-Y-N-G-F-G-N-D-G-S
121	E 1	1420.7	G-D-G-Y-N-G-F-G-N-D-G-S-N-F
122	E 2	1362.7	G-Y-N-G-F-G-N-D-G-S-N-F-G-G
123	E 3	1256.6	N-G-F-G-N-D-G-S-N-F-G-G-G-G
124	E 4	1335.7	F-G-N-D-G-S-N-F-G-G-G-G-S-Y
125	E 5	1360.6	N-D-G-S-N-F-G-G-G-G-S-Y-N-D
126	E 6	1335.7	G-S-N-F-G-G-G-G-S-Y-N-D-F-G
127	E 7	1468.8	N-F-G-G-G-G-S-Y-N-D-F-G-N-Y
128	E 8	1435.7	G-G-G-G-S-Y-N-D-F-G-N-Y-N-N
129	E 9	1536.7	G-G-S-Y-N-D-F-G-N-Y-N-N-Q-S
130	E10	1623.7	S-Y-N-D-F-G-N-Y-N-N-Q-S-S-N
131	E11	1577.7	N-D-F-G-N-Y-N-N-Q-S-S-N-F-G
132	E12	1576.8	F-G-N-Y-N-N-Q-S-S-N-F-G-P-M
133	E13	1557.8	N-Y-N-N-Q-S-S-N-F-G-P-M-K-G
134	E14	1451.7	N-N-Q-S-S-N-F-G-P-M-K-G-G-N
135	E15	1427.8	Q-S-S-N-F-G-P-M-K-G-G-N-F-G
136	E16	1425.9	S-N-F-G-P-M-K-G-G-N-F-G-G-R
137	E17	1398.9	F-G-P-M-K-G-G-N-F-G-G-R-S-S
138	E18	1348.8	P-M-K-G-G-N-F-G-G-R-S-S-G-P
139	E19	1340.8	K-G-G-N-F-G-G-R-S-S-G-P-Y-G
140	E20	1269.7	G-N-F-G-G-R-S-S-G-P-Y-G-G-G
141	E21	1283.7	F-G-G-R-S-S-G-P-Y-G-G-G-G-Q
142	E22	1389.8	G-R-S-S-G-P-Y-G-G-G-G-Q-Y-F
143	E23	1375.8	S-S-G-P-Y-G-G-G-G-Q-Y-F-A-K
144	E24	1454.9	G-P-Y-G-G-G-G-Q-Y-F-A-K-P-R
145	E25	1542.9	Y-G-G-G-G-Q-Y-F-A-K-P-R-N-Q
146	E26	1436.8	G-G-G-Q-Y-F-A-K-P-R-N-Q-G-G
147	E27	1542.9	G-Q-Y-F-A-K-P-R-N-Q-G-G-Y-G
148	E28	1501.9	Y-F-A-K-P-R-N-Q-G-G-Y-G-G-S
149	E29	1365.7	A-K-P-R-N-Q-G-G-Y-G-G-S-S-S
150	E30	1340.6	P-R-N-Q-G-G-Y-G-G-S-S-S-S-S
151	F 1	1337.6	N-Q-G-G-Y-G-G-S-S-S-S-S-S-Y
152	F 2	1239.6	G-G-Y-G-G-S-S-S-S-S-S-Y-G-S
153	F 3	1338.7	Y-G-G-S-S-S-S-S-S-Y-G-S-G-R
154	F 4	1421.8	G-S-S-S-S-S-S-Y-G-S-G-R-R-F
155	F 5	0	
156	F 6	0	
157	F 7	0	
158	F 8	0	
159	F 9	1313.8	A-K-R-R-R-L-S-S-L-R-A
160	F10	0	
161	F11	0	
162	F12	0	
163	F13	0	
164	F14	1313.8	A-K-R-R-R-L-S-S-L-R-A
165	F15	0	
166	F16	0	
167	F17	0	
168	F18	0	
169	F19	0	
170	F20	0	
171	F21	0	
172	F22	0	
173	F23	1313.8	A-K-R-R-R-L-S-S-L-R-A
174	F24	0	
175	F25	0	
176	F26	0	
177	F27	0	
178	F28	1313.8	A-K-R-R-R-L-S-S-L-R-A
179	F29	0	
180	F30	0	

Figure S6

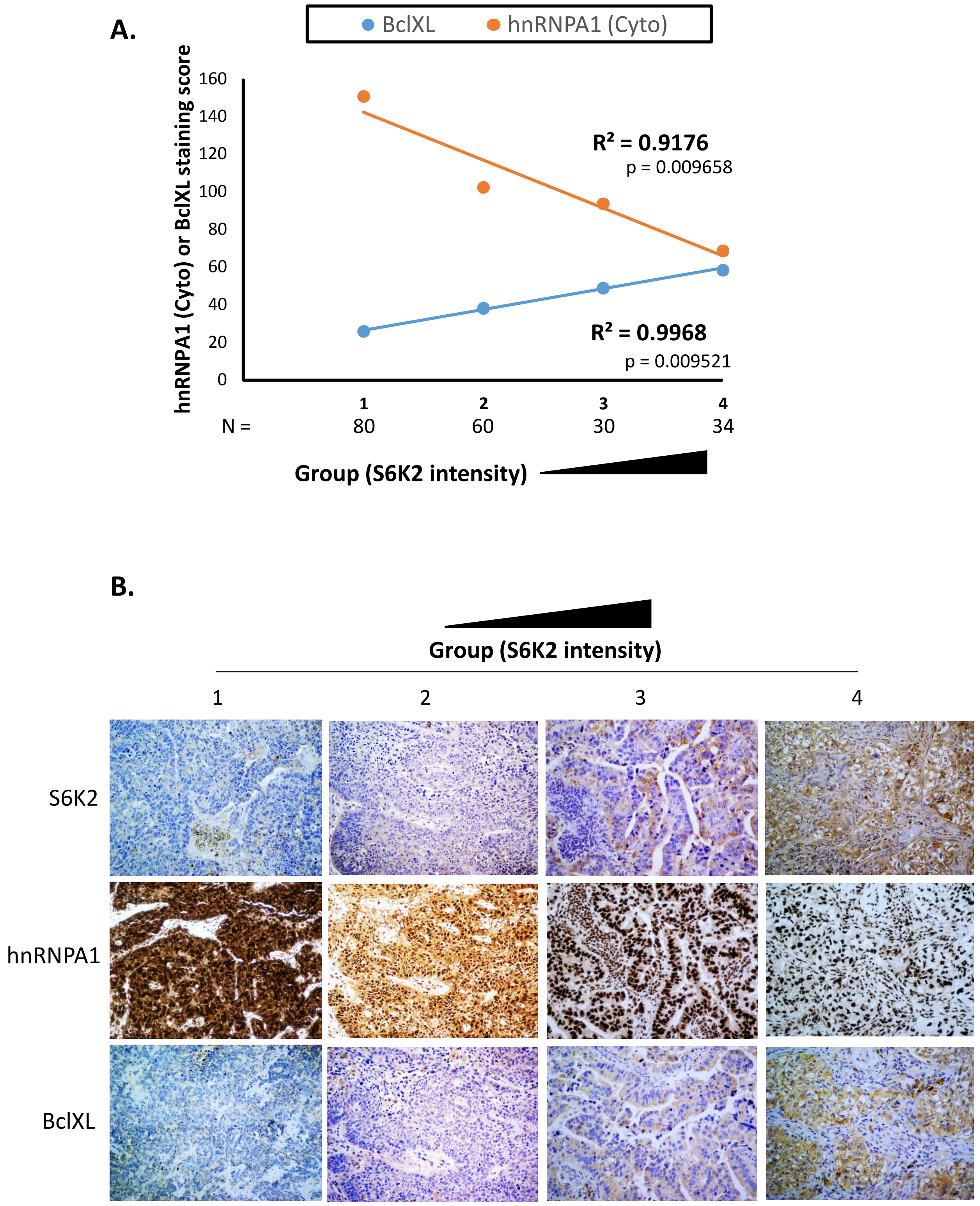

Score				
S6K2	0	30	53	167
A1 (cyto)	300	180	33	0
BclXL	0	30	80	100

Figure S7

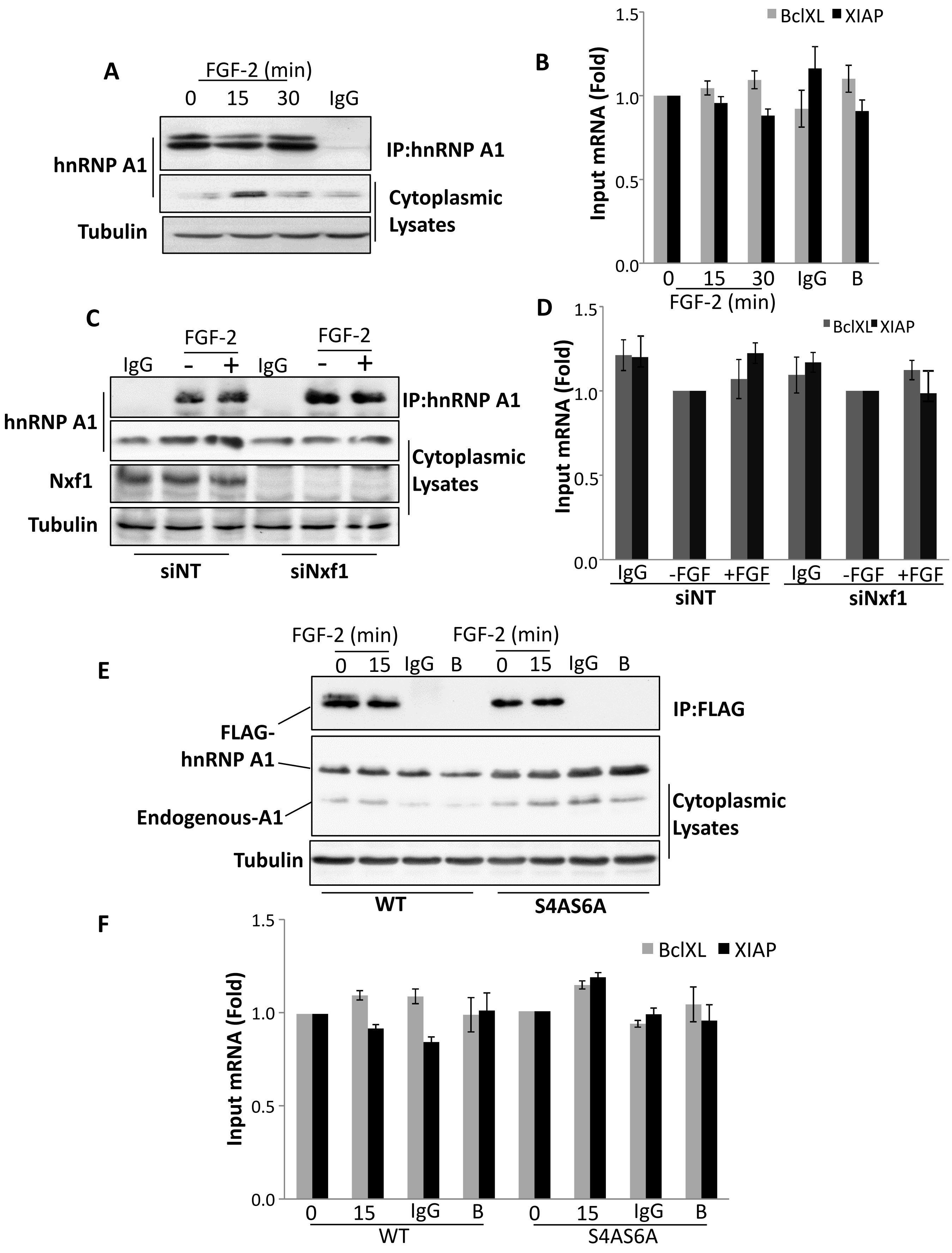
A
FGF-2 (min)
0
15
30
IgG
hnRNP A1
IP:hnRNP A1
Cytoplasmic Lysates
Tubulin
B
BclXL
XIAP
Input mRNA (Fold)
1.5
1.0
0.5
0.0
0
15
30
IgG
B
FGF-2 (min)
C
FGF-2
IgG
-
+
IgG
-
+
hnRNP A1
IP:hnRNP A1
Nxf1
Cytoplasmic Lysates
Tubulin
siNT
siNxf1
D
BclXL
XIAP
Input mRNA (Fold)
IgG
-FGF
+FGF
siNT
siNxf1
E
FGF-2 (min)
0
15
IgG
B
IP:FLAG
FLAG-hnRNP A1
Endogenous-A1
Cytoplasmic Lysates
Tubulin
WT
S4AS6A
F
BclXL
XIAP
Input mRNA (Fold)
0
15
IgG
B
WT
S4AS6A
