# Supplementary figures and images for: Correction to ‘hnRNPA1 couples nuclear export and translation of specific mRNAs downstream of FGF-2/S6K2 signalling’
Source: Nucleic Acids Res. 2024 Jun 14;52(13):8034. doi: 10.1093/nar/gkae525 (PMC11260462; doi:10.1093/nar/gkae525)

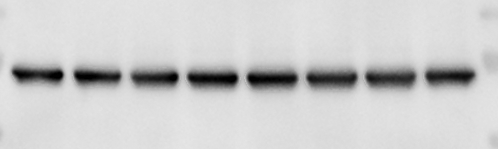

Supplement: gkae525_Supplemental_Files [file gkae525_supplemental_files.zip › 6A-Lamin-nuc fraction.tif]

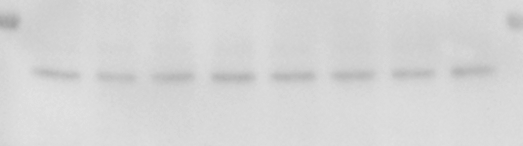

Supplement: gkae525_Supplemental_Files [file gkae525_supplemental_files.zip › 6A-Tubulin-nuc fraction.tif]

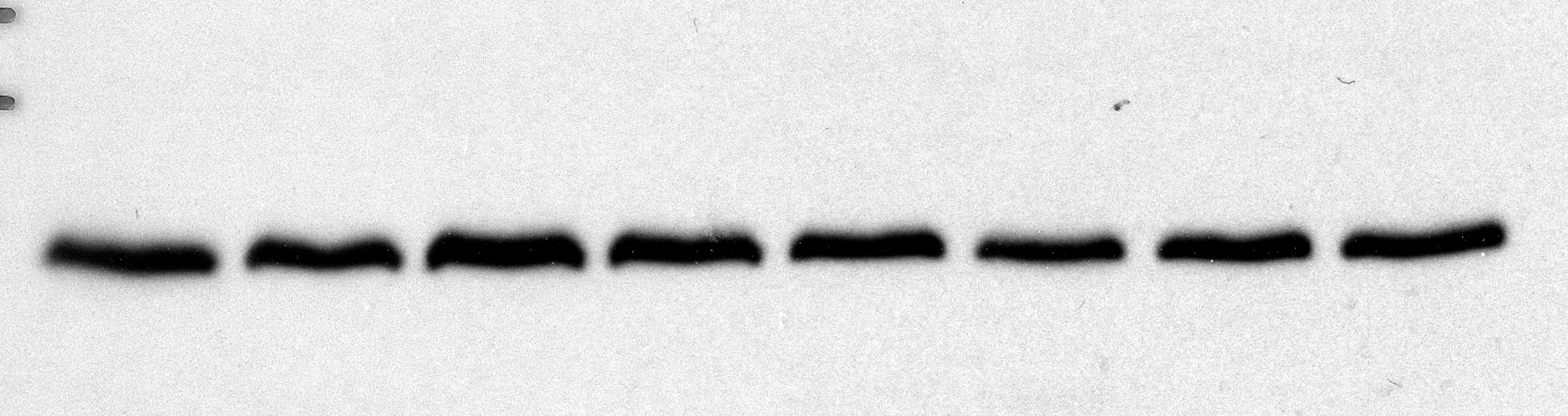

Supplement: gkae525_Supplemental_Files [file gkae525_supplemental_files.zip › 6h-Lamin-nuc fraction.tif]

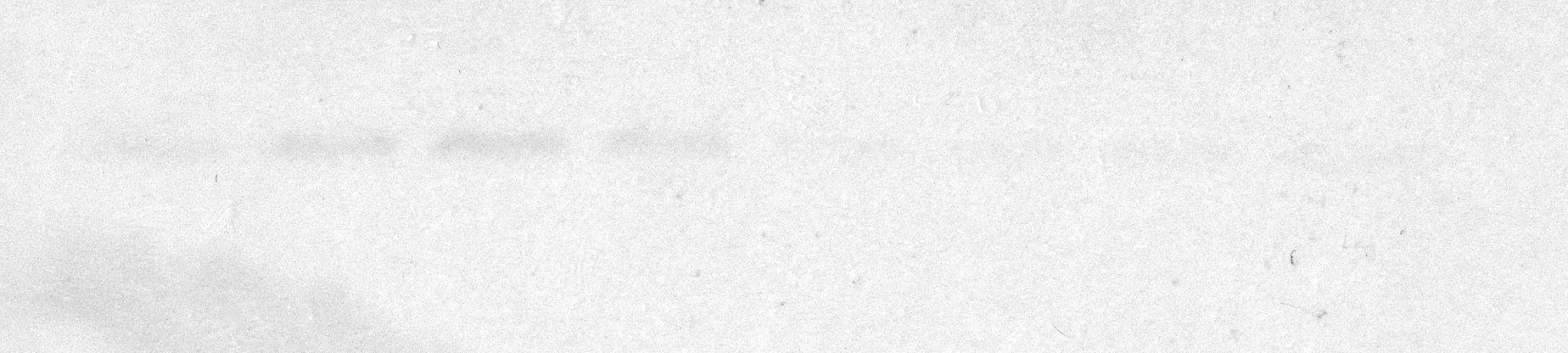

Supplement: gkae525_Supplemental_Files [file gkae525_supplemental_files.zip › 6h-tubulin-nuc fraction.tif]
